# Sparse coding reveals greater functional connectivity in female brains during naturalistic emotional experience

**DOI:** 10.1371/journal.pone.0190097

**Published:** 2017-12-22

**Authors:** Yudan Ren, Jinglei Lv, Lei Guo, Jun Fang, Christine Cong Guo

**Affiliations:** 1 School of Automation, Northwestern Polytechnical University, Xi’an, Shaanxi, China; 2 QIMR Berghofer Medical Research Institute, Herston, Queensland, Australia; Shanghai Maritime University, CHINA

## Abstract

Functional neuroimaging is widely used to examine changes in brain function associated with age, gender or neuropsychiatric conditions. FMRI (functional magnetic resonance imaging) studies employ either laboratory-designed tasks that engage the brain with abstracted and repeated stimuli, or resting state paradigms with little behavioral constraint. Recently, novel neuroimaging paradigms using naturalistic stimuli are gaining increasing attraction, as they offer an ecologically-valid condition to approximate brain function in real life. Wider application of naturalistic paradigms in exploring individual differences in brain function, however, awaits further advances in statistical methods for modeling dynamic and complex dataset. Here, we developed a novel data-driven strategy that employs group sparse representation to assess gender differences in brain responses during naturalistic emotional experience. Comparing to independent component analysis (ICA), sparse coding algorithm considers the intrinsic sparsity of neural coding and thus could be more suitable in modeling dynamic whole-brain fMRI signals. An online dictionary learning and sparse coding algorithm was applied to the aggregated fMRI signals from both groups, which was subsequently factorized into a common time series signal dictionary matrix and the associated weight coefficient matrix. Our results demonstrate that group sparse representation can effectively identify gender differences in functional brain network during natural viewing, with improved sensitivity and reliability over ICA-based method. Group sparse representation hence offers a superior data-driven strategy for examining brain function during naturalistic conditions, with great potential for clinical application in neuropsychiatric disorders.

## Introduction

Functional neuroimaging techniques, such as functional magnetic resonance imaging (fMRI), are widely used to examine changes in brain function associated with age, gender, and a wide range of neuropsychiatric disorders [1–3]. Most of these studies used task-based paradigms, where participants perform laboratory-designed tasks in the scanner. While these tasks are designed to engage and isolate a particular aspect of brain function such as working memory or visual perception, it is unclear whether and to what extent such paradigms could uncover the complex mental processes in real life. To address such limitation, recent fMRI studies employed naturalistic stimuli, such as movie and music, to examine neural processes under real-life condition [4–10]. Despite the dynamic and complex nature of these naturalistic paradigms, they evoke highly consistent brain responses across individuals, laying down the foundation of using naturalistic paradigms to study real-life cognition [4–10].

While naturalistic fMRI paradigms are increasingly used to map brain function in healthy populations, only a handful of studies have adopted them to the examination of group differences [2,7–9]. One challenge is the lack of effective statistical models to decode neural correlates of naturalistic stimuli [11]. The inputs in naturalistic paradigms are often dynamic and complex, hence difficult to model using traditional hypothesis-driven methods such as general linear model (GLM) [12]. Data-driven approaches, which do not depend on specifications of the input stimuli, are hence better suited for naturalistic fMRI studies. Progress has been made with inter-subject correlation and independent component analysis (ICA): several studies have demonstrated that neural synchrony, as measured by inter-subject correlation, during natural viewing is reduced in patients with autism and depression [8,9]. However, inter-subject correlation compares neural responses at the group level, offering very limited view on individual brain responses. ICA, on the other hand, could reconstruct the functional networks for individual brain. A recent naturalistic fMRI study has successfully used ICA to capture altered functional brain networks in individual patients with reduced levels of consciousness [13].

ICA is based on the assumption of independence between each signal source [14]. Since brain is composed of complex interconnected networks, there is no biological reason for different spatial components to hold independent distributions [15]. In addition, ICA does not account for the intrinsic sparsity of neural coding [14]: the brain encodes information with activities of sparse sets of neuronal ensembles while the majority of neurons are silent [16]. To address this issue, recent studies have decomposed fMRI signals into linear combinations of multiple atoms based on sparse representation of whole-brain fMRI signals [17–23]. Sparse population coding of a set of neurons has been showed to be more effective than ICA in reconstructing brain networks [15], which is increasingly applied to fMRI data analyses [18,20,24]. The basic sparse representation pipeline is to extract the whole-brain fMRI signals of one subject into a big data matrix, which is subsequently decomposed into a dictionary matrix and an associated coefficient matrix by sparse coding algorithm [25]. Thus, the time series of each dictionary atom corresponds to the functional activities of a brain network, and its associated coefficient vector represents the spatial map of this brain network.

However, as there is no correspondence of dictionary atoms across subjects and groups, it is difficult to derive group inference or compare group differences using previous single subject sparse representation. To address this problem, we here adopted a group sparse representation-based computational framework to extract functional networks during natural viewing [26]. The advantage of our method is that a common signal dictionary can be learned from the aggregated fMRI signals of two groups of subjects and then the coefficient matrices corresponding to each common dictionary can be used to statistically assess group differences. To learn a common signal dictionary for two groups of subjects from group sparse representation, we assumed that all the subjects evoke highly consistent neural temporal responses during natural viewing, as revealed by previous naturalistic fMRI studies [6,27,28]. Here, we applied group sparse representation to naturalistic fMRI data acquired from healthy males and females while they watched an emotional movie. We then statistically compared the gender differences of functional activity based on the correspondences established by the common learned dictionary. The effectiveness and the reproducibility of our method were further evaluated against ICA-based method. Our results demonstrated the feasibility and superiority of group sparse representation on elucidating functional networks and group differences in functional brain activity under naturalistic conditions.

## Materials and methods

### Overview

**Fig 1:**
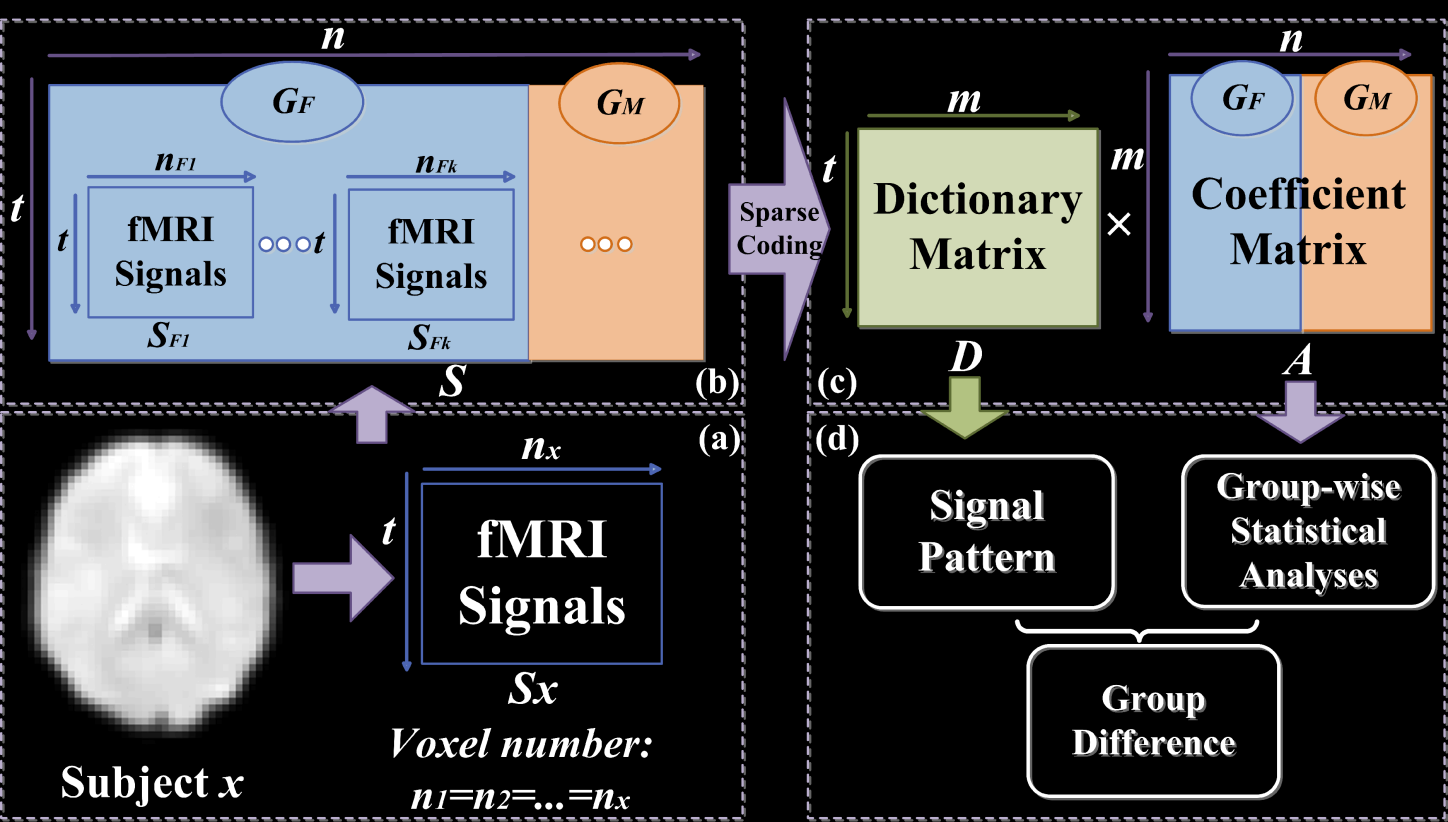
The computational framework of group sparse representation on the whole-brain fMRI signals from two groups of subjects (G_F_: Female, G_M_: Male). (a) Extracting whole-brain fMRI data from subjects ***x*** (subscript represents the label of subject, e.g., ***x***). (b) FMRI data matrices (***S***_***x***_) from all the subjects are aggregated (**S**). (c) Coefficient matrix **A** with the same spatial information and group correspondence of ***S***, which is decomposed into 2 matrices (**A**_**GF**_, **A**_**GM**_) corresponding to two groups (G_F_, G_M_), and each group is made of sub-matrices corresponding to the sparse representation for each subject.

The overview of our analytical pipeline is shown in Fig 1. First, whole-brain fMRI signals of each subject were extracted using a common mask and then stacked into a 2D signal matrix (Fig 1a). Signal matrices from all participants were pooled and concatenated in the spatial dimension into a 2D matrix **S** (Fig 1b), which was then factorized into one common dictionary matrix **D**, and the associated coefficient matrix **A** composed of 2D individual coefficient sub-matrices for each participant, using online dictionary learning and sparse coding method (Fig 1c) [25]. Finally, the derived coefficient matrices were used to assess functional differences between two groups of subjects, and the test-retest reliability of group sparse representation (Fig 1d). All variables used in the main text are defined in S1 Table.

### Data acquisition and pre-processing

18 right–handed (10 females, 8males) healthy subjects (ages 27±2.7) participated in the study, who were all recruited from the University of Queensland and compensated for their participation. Every participant signed a written informed consent. The subject recruitment for the first session lasted from April to October in 2014, while the recruitment for second session lasted from July to December in 2014. The study was approved by the ethics committee of the University of Queensland and was conducted according to National Health and Medical Research Council guidelines. The experiments comprised two scanning sessions with an interval of around 3 months. For each session, participants underwent an 8 min resting state fMRI exam with eyes closed, and then freely viewed a 20-min movie *The Butterfly Circus*. This is a short, positively valenced movie that depicts the story of a man born without limbs who is encouraged by the showman of a renowned circus to discover his own potential. All participants reported that they had not previously seen the movie. The movie stimulus was presented using the Presentation software (NeuroBehavioral Systems, USA) and displayed via an MRI-compatible monitor located at the rear of the scanner. The soundtrack of the movie was delivered through MRI-compatible audio headphones (Nordic NeuroLab, Norway). After 3 months, 16 (9 females, 7 males) subjects were scanned with the same protocol for test-retest reliability analysis (session B dataset), although 2 subjects could not return for the re-scan. Thus these 2 subjects were excluded from our test-retest reliability analysis. All structural and functional images were scanned in a whole-body 3T Siemens Trio MRI Scanner. The scanning parameters are TR/TE/FA/FOV of 2200ms/30ms/79°/134mm×134mm, resolution of 3mm×3mm×3mm, and dimension of 64×64×44.

Functional images were preprocessed using Statistical Parametric Mapping toolbox (SPM12). The preprocessing pipeline included slice timing correction and realigning, co-registration, normalization, motion correction, spatial smoothing with 6mm full width half maximum Gaussian kernel, and band pass filtering (0.0085–0.15 Hz). Nuisance covariates including WM, CSF and Friston-24 motion parameters were then regressed out using the Data Processing Assistant for Resting-state fMRI software (DPARSF) to reduce potential effects of physiological confounds. By computing the intersection of all single brain masks of each participant together, we generated a group-wise common mask to extract whole brain signals. In this way, we ensured that the same voxels are processed for each participant. We also calculated the mean head motion parameter as the mean absolute displacement of each brain volume as compared to the previous volume estimated from the translation parameters in the x (left/right), y (anterior/posterior), and z (superior/inferior) directions (displacement = square root (x^2^+y^2^+z^2^)) of two groups [29], and found no significant group differences of mean head motion parameter (*P*>0.05, Mean±SD: 0.0552 mm±0.0357 for the female group, and 0.0703 mm±0.0292 for the male group).

While the whole naturalistic fMRI dataset was 20-minitute, we first segmented the 20-min fMRI dataset into three segments, according to the narrative structure of the movie *The Butterfly Circus* performed by three experts trained in screenwriting and film theory [10]. We then applied our framework to one segment of fMRI data (7~13min, both sessions) that comprised a complete event, for the efficiency of computation and investigation of the over-completion problem. We finally replicated all analyses on the whole 20-minute dataset for further validation. Based on both results, we investigated the over-completion problem, that is, less observations (n) than predictors (p) [25]. Since the two datasets yielded similar results, only results based on middle segment of fMRI are presented in the main text, while the results based on whole fMRI datasets are provided in the supplemental materials (S1–S4 Figs, S2 and S3 Tables).

#### Affective ratings of the movie

After participants completed each scanning session, they were asked to rate their experience when watching the movie, including the level of boredom, enjoyment, valence, as well as the audio and video quality of the movie during fMRI acquisition, in the scale between 1 and 5 (S4 Table). Note that higher rating of boredom means more boring the participants feel, while higher rating of enjoyment refers to more enjoyable the participants experience. Participants rated the movie as positive and happy (3.7±1.2). For ratings of boredom, where males (2±1.07) tend to rate higher scores than females (1.10±0.32) indicating that females were more engaged than males during movie viewing (*P*<0.0217, two-sample t-test; Table 1). We did not detect significant group differences in ratings of enjoyment, valence and audio and video quality between male and female (*P*>0.05; Table 1).

**Table 1:** Affective ratings of the movie and head motion parameter of females and male.

gender	boredom *	enjoyment	valence	audio/video quality	head motion
female	1.10±0.32	4.20±1.23	3.60±1.17	3.90±0.99	0.0552±0.0357
male	2.00±1.07	3.75±0.89	3.62±1.19	4.00±0.76	0.0703±0.0292

* indicates significant differences between females and males; *P*<0.0217, two-sample t-test

### Dictionary learning and sparse representation theory

The sparse coding framework is implemented by custom codes in Matlab (MathWorks, MA, USA) and a publicly available software (http://spams-devel.gforge.inria.fr/). In this framework, each signal sample s_i_ in the data matrix **S** = [s_1_,s_2_,…,s_n_]∈R^t×n^ is modeled as the sparse and linear combination of atoms in a learned dictionary **D** = [d_1_,d_2_,…,d_m_]∈R^t×m^, i.e., s_i_ = **D** × a_i_ and **S** = **D** × **A**, where **A** = [a_1_,a_2_,…,a_n_]∈R^m×n^ is the weight coefficient matrix for sparse representation and each column a_i_ is the corresponding weight vector for s_i_ [30].

Training a solution for sparse representation of **S** = [s_1_,s_2_,…,s_n_]∈R^t×n^, the empirical cost function is summarized in Eq (1) by considering the average loss of representation for n signals.

$$f_{n}(D)≜\frac{1}{n}\sum_{i=1}^{n}\ell (s_{i},D)$$ (1)

The loss function is defined in Eq (2) with the ℓ_2_ norm that yields the minimization of the representation error, and the ℓ_1_ norm that constrains the sparsity of a_i_. Here, **λ** is a regularization parameter to trade off the representation error and sparsity level.

ℓ(si,D)≜12‖si−Dai‖22+λ‖ai‖1ai∈Rmmin (2)

As we mainly focus on the fluctuation shapes of input signals and aim to prevent **D** from becoming arbitrarily large, we constrain columns d_1_,d_2_,…,d_m_ in **D** with Eq (3).

$$C≜\{D\in R^{t\times m}\;s.t.\;\forall j=1,\ldots m,d_{j}^{T}d_{j}\leq 1\}$$ (3)

In summary, the sparse representation problem is summarized as a matrix factorization problem as shown in Eq (4). Similar as in Eq (2), the Frobenius norm is employed for factorization error minimization, and the ℓ_1_ norm of A matrix yields sparsity. The alternative optimization strategy [31] is usually employed to solve the problem, where the dictionary **D** and coefficient **A** are iteratively optimized, by alternatingly minimizing over one while keeping the other fixed, and the dictionary **D** is initialized randomly, as proposed by [31]. A method based on this strategy, called online dictionary learning, which could deal with infinite data input [25], was proposed and the software (http://spams-devel.gforge.inria.fr/) was developed for public use. Comparing with classical dictionary learning methods that access the whole training data at each iteration to optimize the dictionary **D** and coefficient **A**, the online dictionary learning improves the efficiency by progressively augmenting the training data [25,32]. In each iteration, the sparse coding and dictionary updating is performed with a subset of the training data based on stochastic optimization. Afterwards, the subset is then augmented with a new training sample, and the optimization is performed again on the new training data with the outcome of the previous iteration as warm restart. The online dictionary learning repeats these iterations until all training data have been adopted, providing an efficient solution with low memory consumption and computational cost as compared to classical dictionary learning methods [25]. In this paper, we employ this online dictionary learning and sparse coding method for our group sparse representation analysis.

12D∈C,A∈Rm×nmin‖S−DA‖F2+λ‖A‖1,1 (4)

### Sparse representation of whole-brain fMRI signals

$$S=[S_{G_{F}},\;S_{G_{M}}],S_{G_{F}}=[S_{F1},S_{F2},\ldots ,S_{\text{Fk}}]{,S}_{G_{C}}=[S_{M1},S_{M2},\ldots ,S_{Ml}]$$ (5)

**Fig 2:**
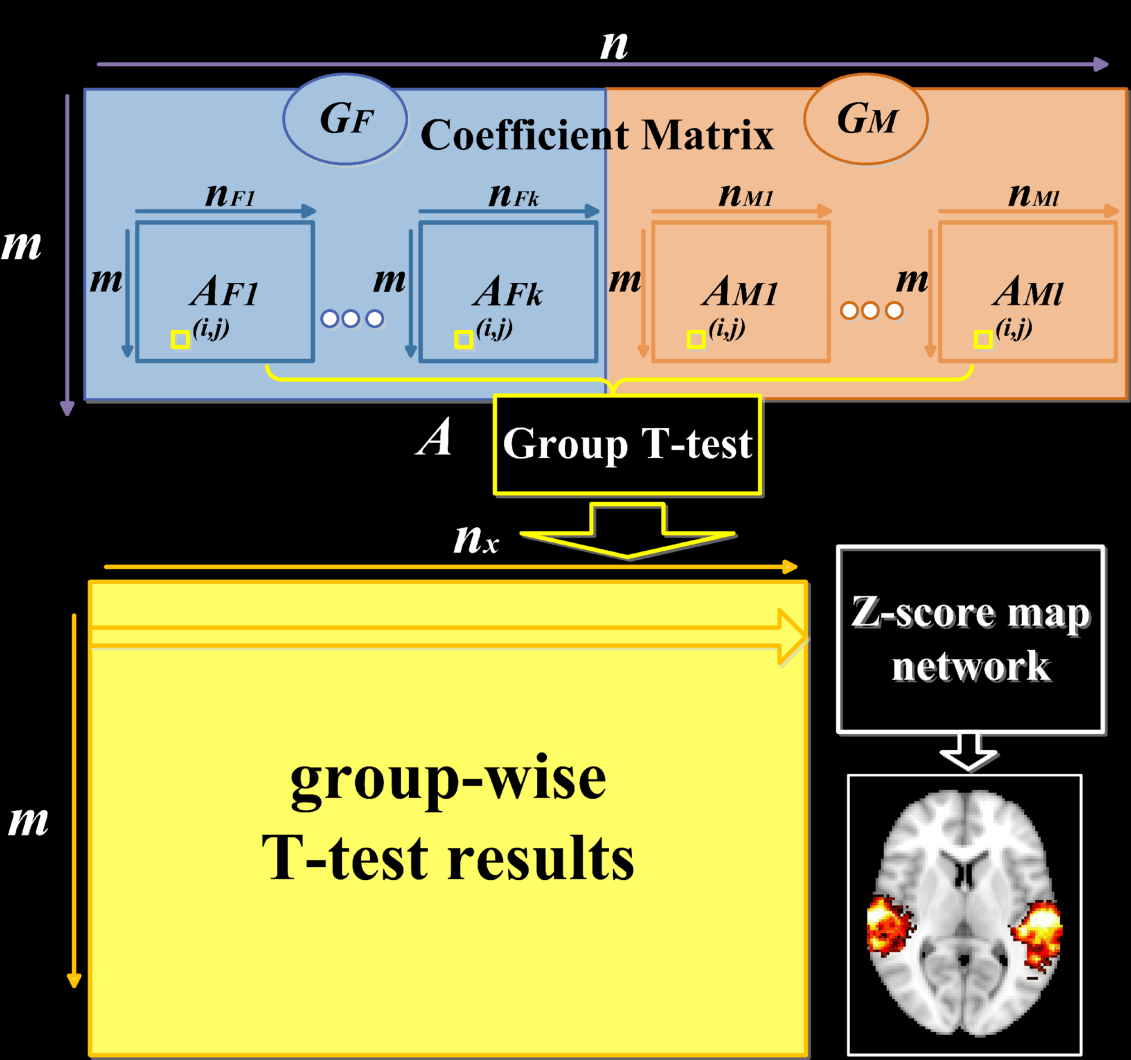
The computational framework of group-wise statistical analysis. Coefficient matrix **A** is composed of two groups of subjects (*G*_*F*_: *k* female subjects, *G*_*M*_: *l* male subjects). Each row in group T-test represents a component network, which is then mapped backed to brain volume color coding with z-scores and called z-score map (***n***_***F1***_ = … = ***n***_***Fk***_ = ***n***_***M1***_ = … = ***n***_***Ml***_ = ***n***_***x***_). Each row of sub-coefficient matrices **A**_***x***_ (**A**_***Fp***_ or **A**_***Mq***_) representing individual coefficient spatial map of all the subjects are set as input of SPM12 for two-sample t-tests.

Whole-brain fMRI signals of each subject were extracted and stacked into a 2D matrix ***S***_***x***_ (***x*** represents the label of participant, ***S***_***Fp***_ or ***S***_***Mq***_). The fMRI signals of each voxel make up the columns of ***S***_***x***_. Then, all signal matrices from two groups were pooled and concatenated in the spatial dimension into a big 2Dmatrix **S** (Fig 1). Online dictionary learning and sparse coding method was adopted to decompose **S** into a learned dictionary matrix **D** and the coefficient matrix **A** [25]. Note that **D** is commonly shared by all subjects, and the **A** has the same spatial voxel organization and group correspondence of **S**, which permits group statistical analyses and comparison. Thus, **A** can be decomposed into 2 matrices which represent male and female groups, and each group comprises sub-matrices of participant as shown in Fig 2, e.g., **A**_***GF***_ is composed of **A**_***F1***_, **A**_***F2***_**… A**_***Fk***_. Each column of D corresponds to a dictionary atom and its time course, and each row in **A**_***x***_ (**A**_***Fp***_ or **A**_***Mq***_) represents its coefficient vector that assigns a coefficient to each voxel in the brain and can be mapped back to the brain volume.

There are two essential parameters in sparse coding strategy: **λ** that keeps balance between the residual error of sparse representation and the sparsity of spatial regions in each atom, and the number of dictionary atoms **m**. Currently, there is no established criteria on determining **λ** and **m**. We thus assessed the impact of parameter settings by systematically varying **λ** (0.1, 0.5, 1) and **m** (100, 200, 400). Specifically, we conducted network decomposition, group difference detection and test-retest reliability analyses using different combinations of **λ** and **m**. Results in the main text were obtained when **λ** is set as 0.5 and **m** as 200, and results using other parameter settings are presented in the supplementary materials. While our main conclusion is relatively robust to different parameter settings (S5–S7 Figs), we found the setting of **λ** as 0.5 and **m** as 200 produces the highest test-retest reliability and highest number of clusters showing significant group difference, suggesting this setting is relatively more robust (S6–S8 Figs).

### Group-wise statistical analysis

$$A=[A_{G_{F}},\;A_{G_{M}}],A_{G_{F}}=[A_{F1},A_{F2},\ldots ,A_{\text{Fk}}]{,\;A}_{G_{C}}=[A_{M1},A_{M2},\ldots ,A_{\text{Ml}}]$$ (6)

Coefficient matrix **A** maintains the spatial information and group correspondence of **S**. **A** can be decomposed into 2 matrices corresponding to two groups (**A**_**GF**_, **A**_**GM**_), and each group is made of sub-matrices corresponding to the sparse representation for each subject (Fig 2). F and M denote female and male, respectively. Each row of this sub-matrix **A**_***x***_ represents the individual coefficient spatial map of each dictionary atom (subscript ***x*** represents the label of participant, e.g., ***Fp*** represents the ***p***th subject in the **F**emale group), so the A_x_(i, j) in each sub-matrix represents the reconstruction coefficient of the j^th^ voxel to the i^th^ atom in the dictionary (Fig 2). For all the subjects together, we hypothesize that each coefficient is group-wisely null, and the T-test (with T defined as Eq (7)) is carried out to test acceptance or rejection of the null hypothesis for each element. The derived T-value is transformed to z-score [12].

$$T(i,j)=\frac{\hat{A_{G}(i,j)}}{\sqrt{Var(A_{G}(i,j))}},\;A_{G}(i,j)={\{A}_{Fp}(i,j):p=1,2,\ldots k;\;A_{Mq}(i,j):q=1,2,\ldots l\}$$

$$\hat{A_{G}(i,j)}=\frac{1}{k+l}(\sum_{p=1}^{k}A_{Fp}(i,j)+\sum_{q=1}^{l}A_{Mq}(i,j))$$

$$Var(A_{G}(i,j))=\frac{1}{k+l}(\sum_{p=1}^{k}{\left(A_{Fp}(i,j)-\hat{A_{G}(i,j)}\right)}^{2}+\sum_{q=1}^{l}{\left(A_{Mq}(i,j)-\hat{A_{G}(i,j)}\right)}^{2})$$ (7)

Due to the sparsity of coefficient matrix **A**, the t-test result of **A** is also sparse. Each row of t-test result represents the statistically significant contribution to each dictionary atom, and can be mapped back to brain volume. The resultant z-score map hence represents the spatial distribution of the atom commonly shared by two groups (Fig 2). Corresponding coefficient spatial maps were then used to compare differences between the two groups.

To test group differences in each dictionary atom, each row of sub-coefficient matrices **A**_***x***_ (***A***_***Fp***_ or ***A***_***Mq***_) are set as input of SPM12 for two-sample t-tests. Search volume is masked by the z-score map from the one-sample t-test corresponding to the same dictionary atom. Both left-tailed and right-tailed two-sample t-tests were performed. Group-difference results were thresholded using a joint probability distribution method to correct for multiple comparisons [33]. Two levels of thresholding were used, where a threshold of *P*<0.005 was set for voxel height, and paired with two different thresholds for cluster extent (FDR-corrected *P*<0.005 and FDR-corrected *P*<0.05).

### Independent component analysis

To evaluate the performance of group sparse representation method, we compared it to three commonly-used data-driven strategies. First, we used tensor independent component analysis (tensor ICA) implemented in FSL MELODIC toolkit [34]. Tensor ICA is commonly used for decomposing the data into independent components where stimulus paradigm is consistent among subjects. Specifically, the number of components in our study was experimentally set to 50, or 100 when specified in the text. Dual regression was then used to project tensor ICA components to each subject space, which were then used for group statistical and test-retest reliability analyses. Similar method is employed to conduct group statistical analysis in SPM12. As no significant results were revealed with the stringent threshold used for group sparse representation, we used a lenient threshold where *P*<0.01was used for voxel height, and *P*<0.01was used for cluster extent.

In addition to tensor ICA, we also examined the functional data with spatial concatenation group ICA method, where the input and output is organized in the same way as group sparse representation [35,36]. This ICA method was implemented using the Fast ICA algorithm [37,38]. Specifically, subjects’ data were concatenated in the spatial dimension into a big signal matrix, which was then factorized into a common time series mixing matrix and the independent spatial components matrix. The spatial components matrix had the same spatial organization of input data and was composed of 2D sub-matrices representing the spatial components for each participant, similar to the coefficient matrix derived by our group sparse representation. Here, the number of components was experimentally set to 50, or 100 when specified in the text. Similar method was adopted to generate group z-score maps and conduct group statistical analysis. As no significant results were revealed with the stringent threshold used for group sparse representation, we used a lenient threshold of *P*<0.01 for voxel height and *P*<0.01 for cluster extent.

Finally, we also adopted commonly used temporal concatenation group ICA, as implemented using GIFT Matlab software [39]. Subject-specific spatial maps were extracted using back-reconstruction for group statistical and test-retest reliability analyses. As no significant results were revealed with the stringent threshold used for group sparse representation, we used a lenient threshold where *P*<0.05 was used for voxel height and *P*<0.05 was used for cluster extent. Since temporal concatenation group ICA yielded similar results, they were mostly presented in the supplemental materials (S9 and S10 Figs, S5 Table).

Corresponding brain networks derived across methods were identified by matching them to established network template [40], followed by careful visual inspection. Furthermore, the corresponding clusters showing gender difference detected across methods were defined by matching them to the Brodmann area and automated anatomical labeling (AAL) atlas, followed by careful visual inspection.

### Test-retest reliability analysis

To test the reproducibility of each brain network fMRI measures, we conducted the same group sparse representation, tensor ICA, spatial and temporal concatenation group ICA on fMRI dataset of session B (16 participants viewed the same movie for the second time), and then identified matching z-score maps which share maximum number of overlapping voxels. The matching was also confirmed with careful visual inspection. For each selected matching network, the networks from two scans were first binarized using a threshold of Z>1.65, and then the common brain region shared by two scans was defined as a mask to evaluate the reliability. The reliability was quantified by calculating the intra-class coefficient (ICC) between measures from the two scans [41,42]. A oneway ANOVA was applied to the measures of the two scans across subjects, to derive between-subject mean square error *MS*_*p*_ and within-subject mean square error *MS*_*e*_, where the measures here referred to z-score, thus ensuring measures of different subjects comparable. ICC values were defined as Eq (8), where *d* is the number of repeated sessions (here *d* = 2). This form of ICC has been widely used in previous test-retest reliability analyses of fMRI data [43,44].

$$ICC=\frac{MSp-MSe}{MSp+(d-1)MSe}$$ (8)

Only the common brain regions shared by both individual spatial maps and mask defined above were included in ICCs calculation. For both group sparse representation and ICA-based methods, we evaluated test-retest reliability at both scan-wise and voxel-wise levels, following previous methods [43]. Scan-wise level was defined as the average z-score across all voxels within the common mask generating a single ICC for whole network, while the voxel-wise level was defined as the individual voxel’s z-score generating ICCs for all the voxels within mask. The test-retest reliability is classified as excellent (ICC>0.8), good (ICC 0.6–0.79), moderate (ICC 0.4–0.59), fair (ICC 0.2–0.39) or poor (ICC<0.2).

## Results

### Functional brain networks identified with group sparse representation

**Fig 3:**
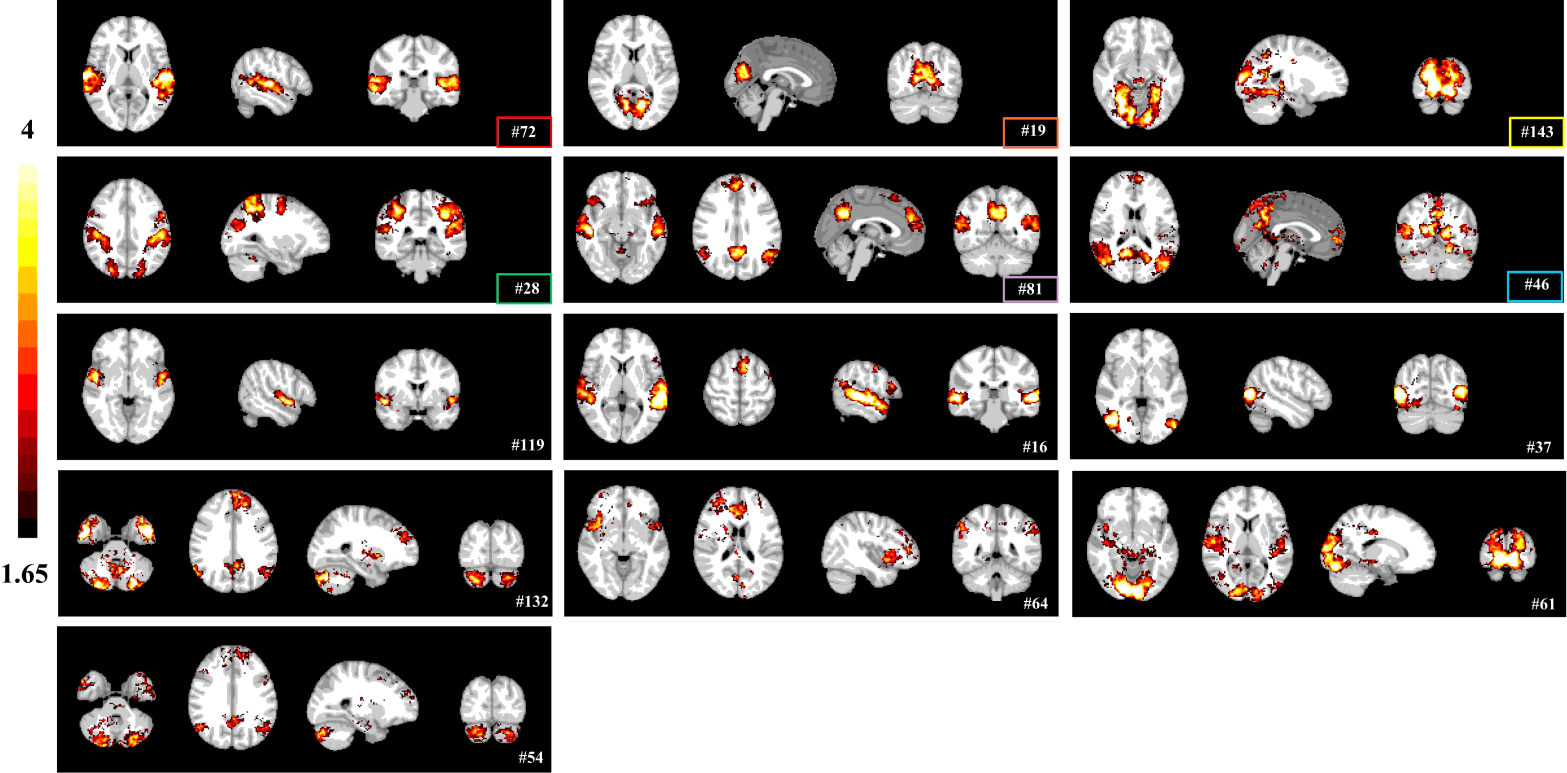
Representative brain networks (z-score maps) identified by group sparse representation method. Networks identified by both sparse representation and ICA methods are highlighted by rectangle frames (color code shared with Fig 4).

We first investigated whether group sparse representation approach could identify functional brain networks of interests during movie viewing. We applied group sparse coding to naturalistic stimuli fMRI data aggregated from 18 healthy subjects. Voxels with significant reference to each dictionary atom were determined using an experimentally determined threshold (Z>1.65, one-sample t-test). Group sparse representation identified several brain networks that have been established previously, including auditory (#72), visual (#19, #143, #37), dorsal attention (#28), default mode (#46), and salience networks (#119) (Fig 3). Some networks identified with sparse representation appeared to be combinations of different networks, such as auditory and supplementary motor networks (#16), default mode and salience networks (#81), default mode and cerebellar networks (#54, #132), salience and executive control networks (#64), auditory and visual networks (#61) (Fig 3). While network patterns identified under different **λ** (0.1, 0.5, 1) and **m** (100, 200, 400) settings are generally similar, the impact of parameter settings can be observed (S5 Fig): the coefficient maps appear to be coarse and noisy with small **λ** and dictionary size, and become sparse with large **λ** and dictionary size (S5 Fig). Overall, the setting of **λ** as 0.5 and **m** as 200 resulted in the most robust network decomposition.

**Fig 4:**
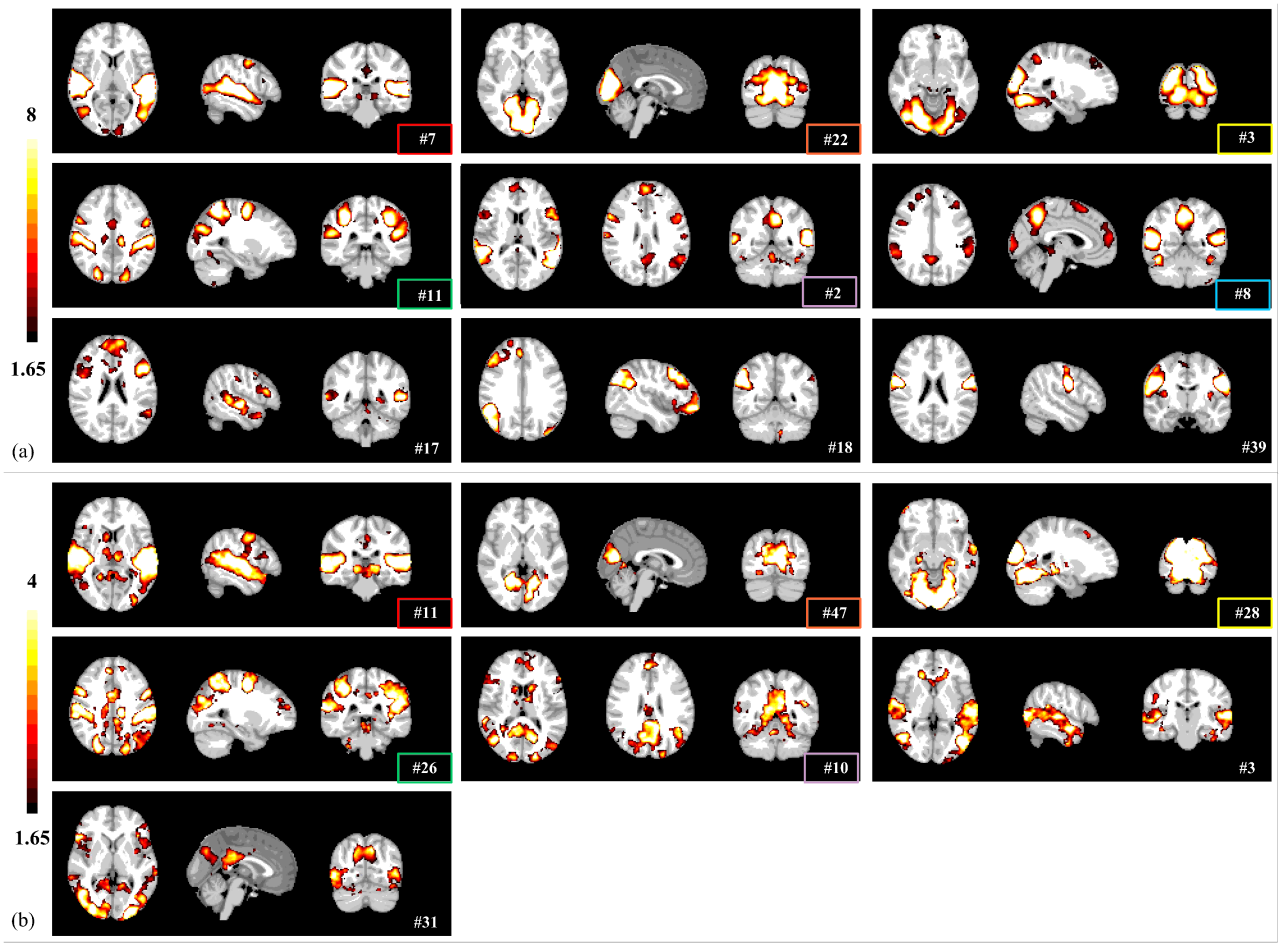
The representative brain networks (z-score maps) identified by (a) tensor ICA method, and (b) spatial concatenation group ICA. Networks identified by both sparse representation and ICA methods are highlighted by rectangle frames (color code shared with Fig 3).

With the same threshold of Z>1.65, both tensor ICA and spatial concatenation group ICA identified similar functional brain networks, including auditory (#7/#11), visual (#3, #22/#28, #47), dorsal attention networks (#11/#26), and default mode and salience (#2/#10) (Fig 4). Five networks were detected by all three methods (labeled in the same color in Figs 3 and 4). These results validate that group sparse representation can identify meaningful brain networks driven by naturalistic stimuli. In addition, sparse representation method detected artifact components that related to head-motion, white matter, susceptibility-motion, cardiac, and MRI acquisition/reconstruction (S11 Fig), similarly to the ICA methods [45,46]. While these networks share spatial patterns with ICA networks in general, they are more circumscribed than the latter, suggesting the sparse representation framework might be more specific and accurate in defining functional networks. We further examined this possibility by comparing 1) the sensitivity in detecting group differences and 2) the test-retest reliability among the three data-driven methods.

### Gender differences in functional brain networks

**Fig 5:**
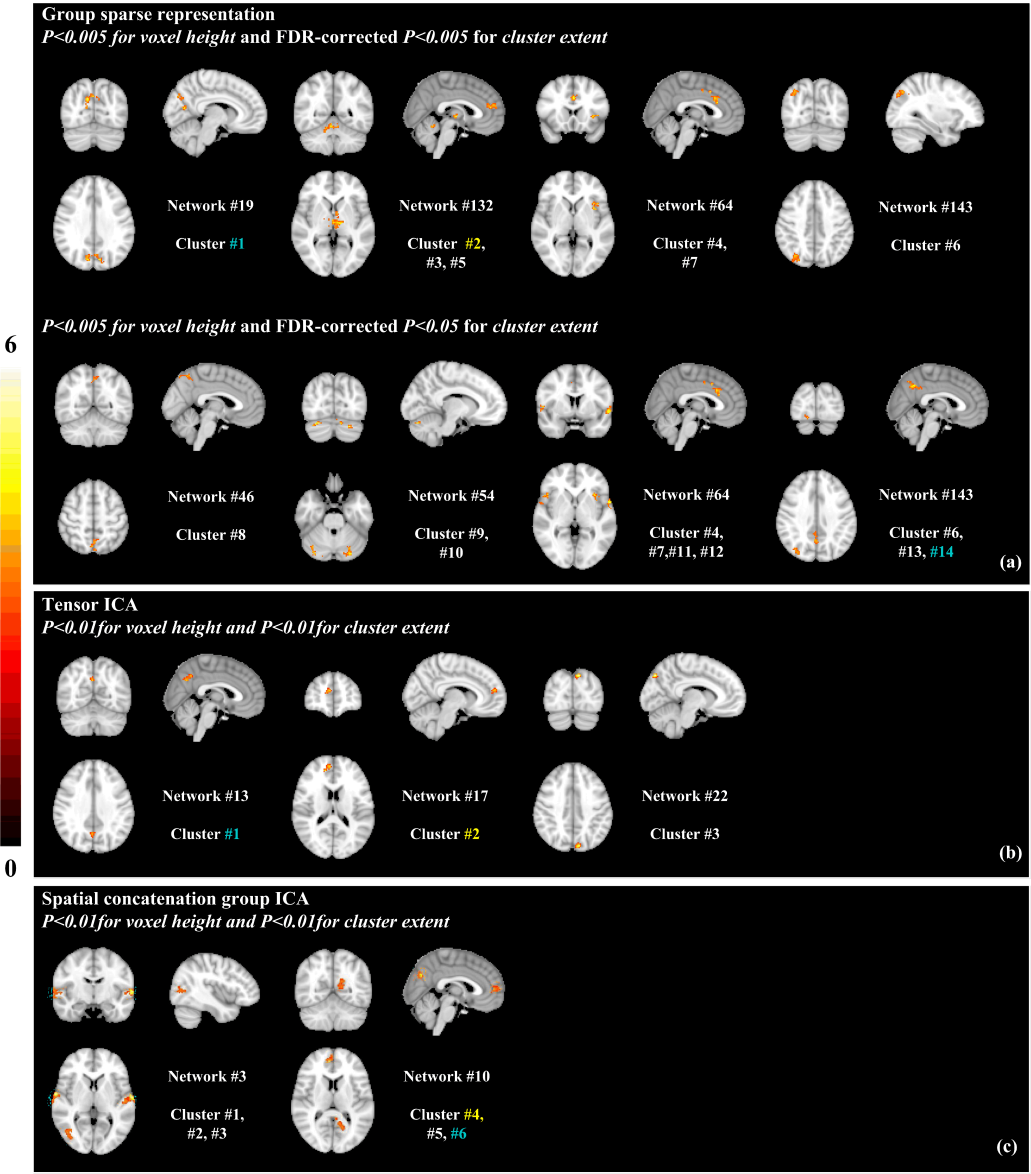
Clusters showing gender difference. (a) 14 clusters detected by group sparse representation that show significantly higher activation in females than males: clusters #1–7 (*P*<0.005 for voxel height and FDR-corrected *P*<0.005 for cluster extent); cluster #8–14 (*P*<0.005 for voxel height and FDR-corrected *P*<0.05 for cluster extent). Clusters detected by (b) tensor ICA and (c) spatial concatenation group ICA that show significantly higher activation in females than males (*P*<0.01 for voxel height and *P*<0.01 for cluster extent). Clusters belonging to same brain region identified by all methods are highlighted in colors.

A question rises up that whether group sparse representation could detect gender differences in brain responses to natural emotional experience. As shown previously, females tend to present greater emotional reactivity [2,7,47,48]. Hence, we hypothesized that brain responses to the naturalistic emotional stimulus is more robust in female than male participants. To test this hypothesis, we compared the coefficient spatial maps for each dictionary atom between the female and male groups. Remarkably, several network regions were found to show higher coefficients, or stronger functional connectivity, in females than males, whereas no regions were found to be higher in males (Fig 5a; two-sample t-tests). Specifically, females display significantly increased functional connectivity in higher order brain centers, including posterior cingulate cortex, precuneus, insula, anterior cingulate cortex, superior medial frontal gyrus, and superior parietal lobule, as well as thalamic nucleus and medial occipital gyrus (Fig 5a; Table 2, clusters 1–7; *P*<0.005 for voxel height and FDR-corrected *P*<0.005 for cluster extent). At a less stringent threshold, additional 7 clusters were detected at the precuneus, posterior cingulate cortex, superior temporal gyrus, inferior frontal gyrus, primary and secondary visual cortices and cerebellar Crus I (Fig 5a; Table 2, clusters 8–14; *P*<0.005 for voxel height and FDR-corrected *P*<0.05 for cluster extent). Note, with this less stringent threshold or even an uncorrected threshold (*P*<0.005 for voxel height and *P*<0.005 for cluster extent), we did not detect any clusters showing greater functional connectivity in male than female participants.

**Table 2:** Brain regions with greater activation in females than males as detected by group sparse representation (sorted by *p*-value in ascending order). The Network Index refers to the index of dictionary atom generated by group sparse representation algorithm (corresponding to the index in Fig 3).

Cluster Index	(x y z)	T-value	Broadmann’s area	Region	Cluster size	Network Index
1	(-10–76 34)	6.45	7,31	precuneus, posterior cingulate cortex	245	19
	(12–72 30)	4.97				
	(12–70 14)	4.60				
2	(2 46 16)	4.74	9, 10, 32	superior medial frontal lobe, anterior cingulate cortex	245	132
	(-10 52 16)	4.58				
	(-4 36 20)	4.30				
3	(-16–20–2)	6.17		medial dorsal nucleus, ventral lateral nucleus	366	132
	(-2–14–2)	5.90				
	(5–20 0)	4.83				
4	(0 20 30)	4.77	32,24	anterior cingulate cortex	180	64
	(-2 12 40)	4.59				
	(6 22 14)	4.15				
5	(12–50–18)	4.71		cerebellum	185	132
	(4–54–14)	4.61				
	(-6–54–16)	4.42				
6	(32–70 40)	4.15	7, 19	superior parietal lobule, medial occipital gyrus	147	143
	(28–78 42)	3.95				
	(38–80 42)	3.18				
7	(-26 26–10)	6.07	13	insula	150	64
	(-32 8 4)	4.92				
	(-32 20 0)	4.32				
8	(0–54 48)	4.24	7	precuneus	104	46
	(2–58 54)	3.83				
	(-2–50 54)	3.80				
9	(26–74–24)	5.13		cerebellum	119	54
	(34–86–32)	4.46				
	(20–80–24)	4.23				
10	(-26–80–30)	5.80		cerebellum	140	54
	(-12–76–24)	4.27				
	(-22–66–32)	4.17				
11	(46 22 2)	4.93	22, 47	superior temporal gyrus, inferior frontal gyrus	95	64
	(54 4 4)	4.07				
	(50 20–6)	4.02				
12	(-58 6–2)	5.25	22	superior temporal gyrus	99	64
	(-58–2 0)	3.48				
	(-50 12–12)	3.11				
13	(20–96–4)	5.19	17, 18	Primary visual cortex, secondary visual cortex	81	143
	(18–92–14)	4.40				
	(10–80–18)	3.97				
14	(2–56 40)	4.48	7, 31	precuneus, posterior cingulate cortex	105	143
	(-4–64 48)	4.23				
	(4–44 36)	3.31				

In contrast, the effect of gender difference was much weaker with tensor ICA and spatial concatenation group ICA networks, where no significant gender difference was detected using either the two FDR-corrected thresholds (*P*<0.005 for voxel height and FDR-corrected *P*<0.005 or 0.05 for cluster extent), or the one without FDR correction (*P*<0.005 for voxel height and *P*<0.005 for cluster extent). Only with a more lenient threshold were we able to detect significant clusters– 3 clusters for tensor ICA and 6 clusters for spatial concatenation group ICA—with greater connectivity in female than male participants (Fig 5b and 5c, S6 Table, clusters 1–3, S7 Table, cluster 1–6; *P*<0.01 for voxel height and *P*<0.01 for cluster extent). Two of these clusters, precuneus and anterior cingulate cortex, were detected with the group sparse representation results at stringent FDR-corrected thresholds (highlighted with same color in Fig 5). These results suggested that group sparse representation is more effective and sensitive for detecting functional differences in brain networks than the ICA-based method.

In addition, we examined whether these results are robust to different parameter settings in sparse representation. We repeated the group statistical comparison analyses with **λ** of 0.1, 0.5, and 1, and dictionary size of 100, 200, and 400. Across all settings, we detected significantly greater functional connectivity in female than male group, and nothing in the opposite contrast (*P*<0.005 for voxel height and FDR-corrected *P*<0.05 for cluster extent). The comparison with **λ** as 0.5 and m as 200 appeared to be the most sensitive, with the more significantly different regions detected than other settings (S6 Fig). Nonetheless, many of the same clusters were detected using different parameters (S7 Fig), suggesting the gender difference results were robust to parameter settings in sparse representation.

### Test-retest reliability

**Fig 6:**
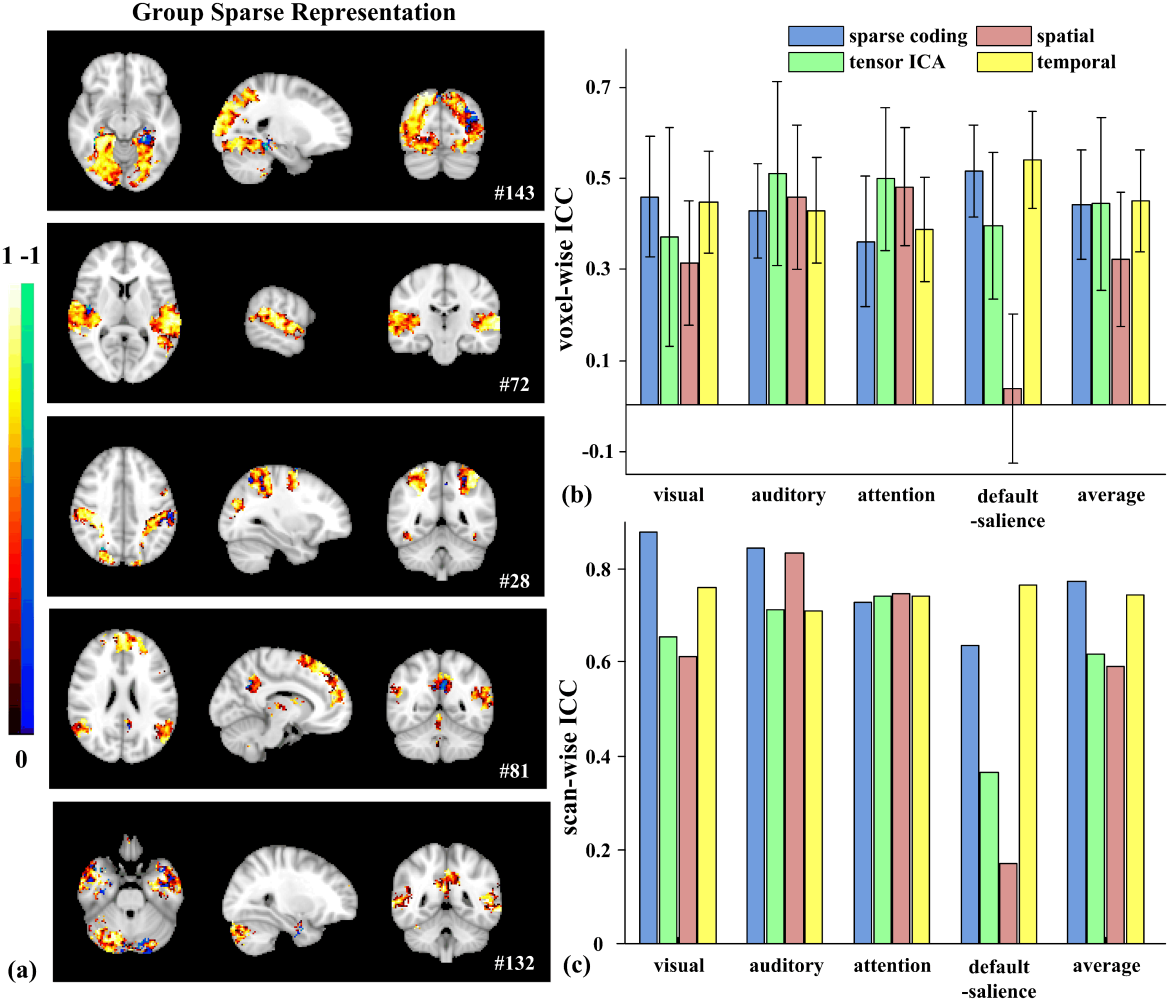
Test-retest reliability of brain networks. (a) Brain maps of the voxel-wise ICCs of matching networks identified by group sparse representation. (b) Average voxel-wise ICCs and (c) scan-wise ICCs of networks detected by all the methods (Sparse representation/tensor ICA/spatial concatenation group ICA/temporal concatenation group ICA: Visual: #143/#3/#28/#30; Auditory: #72/#7/#11/#1; Dorsal attention: #28/#11/#26/#32; Default mode-salience: #81/#2/#10/#11). Error bars signify variance.

Next, we sought to establish the long term test-retest reliability of our method. The same participants underwent the fMRI scan while they viewed the same movie three months later (session B). Group sparse representation, tensor ICA, spatial and temporal concatenation group ICA were used to identify functional brain networks in the repeated scan sessions. To compare the test-retest reliability of these methods, we focused on four networks that can be detected between session A and B by all three methods: visual network, auditory network, dorsal attention network, and default mode-salience network (Fig 6 and S8 Fig). In addition to these four networks, group sparse representation identified one more matching network, the default mode-cerebellar network (Fig 6a), while tensor ICA identified the frontoparietal network.

The reliability was assessed at both voxel-wise and scan-wise levels [43]. All four methods showed a range of reliability at the voxel level across different networks (Fig 6), with greater variability in tensor ICA networks (larger error bars in Fig 6b). On average, voxel-wise ICCs are moderate in the visual network (0.4573, #143; 0.4459, #30) and the default mode-salience network (0.5147, #81; 0.5376, #11) using group sparse representation and temporal concatenation group ICA, but reduced to fair or poor level with tensor ICA (0.3693, #3; 0.3940, #2) and spatial concatenation group ICA (0.3131, #28; 0.0363, #10) (Fig 6b). For the auditory network, average voxel-wise ICCs are at the moderate level using all four methods (0.4261, #72; 0.5086, #7; 0.4558, #11; 0.4268, #1) (Fig 6b). Average voxel-wise ICCs are fair in the dorsal attention network using group sparse representation (0.3595, #28) and temporal concatenation group ICA (0.3869, #32), and are at moderate level using the other two ICA methods (0.4969, #11 and 0.4794, #26) (Fig 6b). To statistically compare the reliability among four methods, we focused on the voxels that are identified by all methods for these matching networks. In two out of four (visual and default mode-salience), voxel-wise ICCs are significantly higher with group sparse representation than tensor ICA and spatial concatenation group methods (paired t-tests, Bonferroni-corrected *P*<0.001, Fig 6b).

Consistent with previous report [43], scan-wise ICCs are improved over the average voxel-wise ICCs (Fig 6b and 6c). Specifically, scan-wise ICCs of the visual and auditory networks using sparse representation are both excellent (0.8786, #143 and 0.8439, #72), and reduced some with tensor ICA (0.6543, #3; 0.7131, #7), spatial (0.6123, #28; 0.8317, #11) and temporal (0.7586, #30; 0.7087, #1) concatenation group ICA (Fig 6c). For the dorsal attention network, scan-wise ICCs are all good across all four methods (0.7269, #28; 0.7402, #11; 0.7452, #26; 0.7418, #32) (Fig 6c). The reliability of the default mode-salience network also shows much higher values with temporal concatenation group ICA (0.7632, #11) and sparse representation (0.6357, #81) than the two ICA methods (0.3634, #2 and 0.1689, #10). Some additional networks identified by sparse representation also showed excellent scan-wise ICC such as the default mode-cerebellar network (0.8064, #132).

To further assess the impact of parameter setting on test-retest reliability, we repeated the reliability analyses using a combination of component numbers and lambda (**m** = 100, 200, 400; **λ** = 0.1, 0.5, 1) for group sparse representation and a high component number for tensor ICA and spatial concatenation group ICA (100) (S8 Fig). We chose three functional networks that can be robustly identified under all settings: visual, auditory and dorsal attention network. Averaging across the three networks, the highest reliability was obtained with **m** = 200 and **λ** = 0.5, further supporting the use of these parameters in the main analyses (S8 Fig). Similarly, tensor ICA and group ICA were more reliable with 50 as the component number than 100 (S8 Fig).

## Discussion

A variety of methods have been developed for functional neuroimaging analysis, such as general linear model (GLM) [12], seed-based method [49], principal component analysis (PCA) [50], singular value decomposition (SVD) [51], inter-subject correlation (ISC) [8], and independent component analysis (ICA) [40]. While GLM is widely used in detecting task-evoked brain activations, ICA is one of the most common methods in characterizing brain network using naturalistic or resting-state fMRI paradigms. Tensor ICA is particularly suitable for naturalistic fMRI paradigms when data are acquired with consistent stimulus presentation [9]. However, the underlying statistical assumption of ICA is not well supported by neurobiological basis—brain networks are not necessarily independent from each other. Rather, they could recruit inputs from the same cortical region and interact between each other in the service of behaviors [15,52–57]. On the other hand, sparse coding algorithm does not constrain dictionary matrix to be uncorrelated or independent, resulting in low level of correlation among atoms [18,24]. In addition, sparse population coding of a set of neurons has been shown to be more effective in detecting brain activity patterns and brain networks [15,17,23]. Recently, a Sparse SPM algorithm based on group sparse dictionary learning shows superior performance for characterizing resting state functional network, over seed-based and ICA methods. However, due to generating common group network and different individual temporal dictionaries, this algorithm differs from our framework that assumes common temporal responses across subjects [23]. Therefore, our group sparse representation framework offers potential advantage in extracting functional brain networks during naturalistic paradigms. Our study presents comprehensive comparisons between group sparse representation-based and three ICA-based methods, based on their performance on network decomposition, group difference detection and test-retest reliability analyses. Our findings suggest that, while all four methods offer comparable test-retest reliability, sparse representation-based method could be more sensitive in identifying functional brain networks during naturalistic paradigms.

Our method builds upon previous studies that employed group sparse representation on static task-based fMRI data [26], and provides one of the first applications of group sparse representation to naturalistic neuroimaging paradigms. Comparing with individual-based sparse representation methods [18,24], group sparse representation method can automatically establish correspondences across individuals and populations, enabling detailed examinations of group differences or brain-behavioral correlations. Our results revealed that this method could identify well-known functional connectivity networks during dynamic naturalistic stimulation. Furthermore, we developed rigorous statistical tests to characterize group differences in functional activity, taking full advantage of the inherent correspondence between individual networks established by group sparse representation.

The current study employed group sparse representation to detect gender differences in brain responses during natural emotional experience. This was motivated by previous findings in psychology and cognitive neuroscience that females respond more strongly than males to affective stimuli [7,47,58–62]. It is well recognized that females responds more strongly than males to affective stimulus, particularly in limbic regions such as the anterior cingulate cortex in response to negative valence [60,62]. Here, we examined the gender differences in emotional experience using a ecologically-valid paradigms [9]. We found support from behavioral ratings that females were more engaged by this dynamic emotional stimulus than males (Table 1). Furthermore, our study identified several brain regions that showed stronger functional activations in female. Many of these brain regions are known to contribute to affective processing. Several clusters are detected at the anterior insula (cluster #7) and anterior cingulate cortex (cluster #2, 4), which coactive in response to emotional salience [63]; the anterior insula is particularly postulated as a hub region that integrates intero- and exteroceptive information and generates the subjective experience of emotion [64,65]. In addition, the default mode network, anchored by the precuneus (cluster #1, 8, 14) and posterior cingulate cortex (PCC) (cluster #1, 14), is activated more strongly in females than males, potentially reflecting greater episodic memory retrieval and self reflection associated with emotion and pain [66,67]. The superior temporal gyrus also showed stronger activations in females than males (cluster #11, 12), consistent with its role in processing emotional facial stimuli [68]. Although the underlying mechanism of these findings requires further investigation, they support greater responses to naturalistic emotional stimuli in females than males.

**Fig 7:**
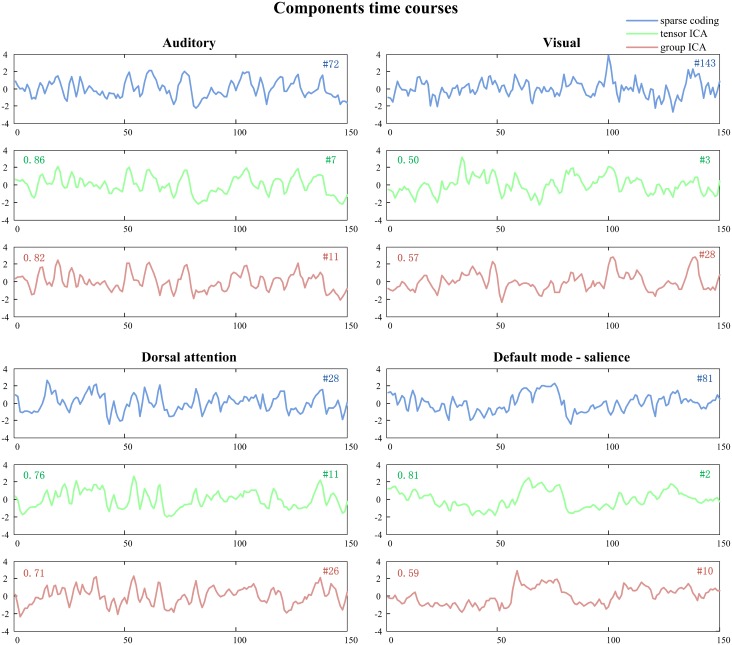
Time courses of representative networks identified by group sparse representation, tensor ICA and spatial concatenation group ICA. Pearson’s correlation between the time courses of group sparse representation and ICA is labeled on the panel of the corresponding ICA method (upper left corner).

Our study not only demonstrated gender differences in naturalistic emotional experience, but also characterized meaningful functional brain networks. As shown in Fig 3, several brain networks identified with group sparse representation are engaged in audio-visual processing, including visual (#19, #143, #37) and auditory networks (#72). In addition, networks well established in the resting state literature are also detected, including the dorsal attention network (#28) associated with attention-demanding activities [69], salience network (#119) responding for salience detection [70], and default mode network (#46) [71]. Interestingly, sparse representation approach also identified brain networks that appear to be combinations of different network regions, such as auditory and supplementary motor networks (#16), default mode and salience networks (#81), default mode and cerebellar networks (#54, #132), salience and executive control networks (#64), auditory and visual networks (#61), which could reflect the interactions between these brain regions during movie viewing. These networks show consistent spatial and temporal patterns across sparse representation and ICA methods (Figs 3, 4 and 7). We then further assessed the test-retest reliability of these brain networks identified by our method. While participants might be less engaged in viewing the same movie for the second time, we still observed good test-retest reliability for several networks, confirming that the natural viewing condition manifests improved reliability over resting state condition [72]. On the other hand, we found that the visual, auditory and attention networks show higher scan-wise ICC values than that of default-salience network (Fig 6). The lower reliable brain activities of higher order brain regions might indicate affective experiences might have changed during repeated viewing. Nonetheless, as our main goal of reliability analysis is to compare the performance of our method to other ICA-based methods, the absolute values for reliability is not the focus of this study. In addition, sparse representation method could separate artifact components relating to head-motion, white matter, susceptibility-motion, cardiac, and MRI acquisition/reconstruction (S11 Fig) [45,46]. Some of these artifact components might also be influenced by the naturalistic stimuli, such as ones related to the head motion. Overall, our method can effectively and robustly detect meaningful brain networks driven by naturalistic stimuli.

We further assessed the effect of parameter setting in sparse representation with rigorous statistical analysis (S6 and S7 Figs). We found that the gender differences in functional connectivity were very robust to parameter settings—significantly greater functional connectivity in female than male group was identified across all parameter settings (*P*<0.005 for voxel height and FDR-corrected *P*<0.05 for cluster extent). The setting used for the main analysis, **λ** as 0.5 and **m** as 200, generated the highest number of clusters showing significant gender difference (S6 Fig). Network components extracted with this setting also showed the highest test-retest reliability (S8 Fig), suggesting the selection of this parameter setting is relatively more robust. Furthermore, our application was not significantly impacted by over-completion, that is, less observations (n) than predictors (p). Here, the observations are time points of fMRI scan, and the predictors refer to the learned dictionary atoms. Sparse representation using either a segment of fMRI data (n<p) or the whole data (n>p) generated very similar results (S1–S4 Figs, S2 and S3 Tables), suggesting that our results are robust to over-completion. Other studies have also shown that over-completion is a valid setting in Lasso framework and appears to offer several advantages, including greater robustness when facing with noises and other kinds of degradation, superior flexibility in matching the generative model to the structure of input data and better approximation of the underlying statistical distribution [25,73–75].

In summary, our study developed a novel application of group sparse representation on naturalistic fMRI data. Using rigorous statistical analyses, we demonstrated that group sparse representation could reliably identify functional networks during natural viewing and detected subtle gender differences in these networks with higher sensitivity than ICA-based method. Hence, the group sparse representation framework could offer a suitable approach in analyzing dynamic fMRI data during naturalistic paradigms. In the future, dimension reduction of input data can be investigated to further improve the effectiveness and efficiency of our framework. In addition, it would be useful to test our framework to naturalistic fMRI datasets acquired during different emotional movies to further validate and investigate gender differences during affective processing. Moreover, this framework could potentially be useful in clinical research, to detect abnormal brain function and develop neuroimaging markers for neuropsychiatric disorders. One potential concern is that if large differences exist between the groups, such as patients and healthy controls, it may be inappropriate to pool fMRI data together for dictionary training. Further investigation is needed to further explore the potential of sparse representation in clinical neuroscience.

## Supporting information

S1 Fig Fifteen clusters detected by group sparse representation for whole fMRI data that show significantly higher activation in females than males. (a) Clusters #1 (*P*<0.005 for voxel height and FDR-corrected *P*<0.001 for cluster extent) (b) Cluster #2–15 (*P*<0.005 for voxel height and FDR-corrected *P*<0.05 for cluster extent). Clusters identified by both group sparse representation and tensor ICA are highlighted in color (color code shared with S2 Fig).
(TIF)

S2 Fig Two clusters detected by tensor ICA for whole fMRI data that show significantly higher activation in females than males (*P*<0.01 for voxel height and *P*<0.01 for cluster extent). Clusters identified by both group sparse representation and tensor ICA are highlighted in colors (color code shared with S1 Fig).
(TIF)

S3 Fig Test-retest reliability of brain networks identified for whole fMRI data. (a) Brain maps of the voxel-wise ICCs of matching networks identified by group sparse representation and tensor ICA for whole fMRI data. (b) scan-wise ICCs and (c) average voxel-wise ICCs of networks detected by both methods. Error bars signify variance.
(TIF)

S4 Fig Comparison of test-retest reliability results using different parameters (m = 100, 200, 400; λ = 0.1, 0.5, 1) for group sparse representation and different component numbers for tensor ICA (m = 50, 200) (whole fMRI data). (a) Brain maps of the voxel-wise ICCs of matching networks identified by the two methods. (b) Average scan-wise and voxel-wise ICCs (Error bars signify the average variance of each network’s voxel-wise ICC).
(TIF)

S5 Fig Seven representative functional networks identified using different parameters (m = 100, 200,400; λ = 0.1, 0.5, 1) for group sparse representation. (TIF)

S6 Fig Comparison of number of clusters showing significantly greater functional connectivity in female than male group using different parameters (m = 100, 200,400; λ = 0.1, 0.5, 1) for group sparse representation. (TIF)

S7 Fig corresponding clusters showing significantly greater functional connectivity in female than male group using different parameters (m = 100, 200, 400; λ = 0.1, 0.5, 1) for group sparse representation (clusters identified under m = 200 and λ = 0.5 share the same indices with Fig 5 in main text). (TIF)

S8 Fig Comparison of test-retest reliability results using different parameters (m = 100, 200, 400; λ = 0.1, 0.5, 1) for group sparse representation, and different component numbers for tensor ICA and spatial concatenation group ICA (m = 50, 100), and temporal concatenation group ICA. (a) Brain maps of the voxel-wise ICCs of matching networks identified by the three methods. (b) Average scan-wise and voxel-wise ICCs (Error bars signify the average variance of each network’s voxel-wise ICC).
(TIF)

S9 Fig The representative brain networks identified by temporal concatenation group ICA. Networks identified by all methods are highlighted by rectangle frames (color code shared with Fig 3 in the main text).
(TIF)

S10 Fig Clusters detected by temporal concatenation group ICA that show significantly higher activation in females than males (*P*<0.05 for voxel height and *P*<0.05 for cluster extent). Clusters identified by all methods are highlighted in colors (color code shared with Fig 5 in the main text).
(TIF)

S11 Fig Artifact related components identified by (a) group sparse representation, (b) tensor ICA, (c) and spatial concatenation group ICA. (TIF)

S1 Table The variables and the explanations. (DOCX)

S2 Table Brain regions with greater activation in females than males as detected by group sparse representation for whole fMRI data(sorted by *p*-value in ascending order). (DOCX)

S3 Table Brain regions with greater activation in females than males as detected by tensor ICA for whole fMRI data(sorted by *p*-value in ascending order). (DOCX)

S4 Table The questionnaire for subjects rating their experience after scanning session. (DOCX)

S5 Table Brain areas with greater activation in females than males as detected by temporal concatenation group ICA (sorted by *p*-value in ascending order). (DOCX)

S6 Table Brain areas with greater activation in females than males as detected by tensor ICA (sorted by *p*-value in ascending order). (DOCX)

S7 Table Brain regions with greater activation in females than males as detected by spatial concatenation group ICA (sorted by *p*-value in ascending order). (DOCX)
