# Supplementary figures and images for: Sparse coding reveals greater functional connectivity in female brains during naturalistic emotional experience
Source: PLoS One. 2017 Dec 22;12(12):e0190097. doi: 10.1371/journal.pone.0190097 (PMC5741239; doi:10.1371/journal.pone.0190097)

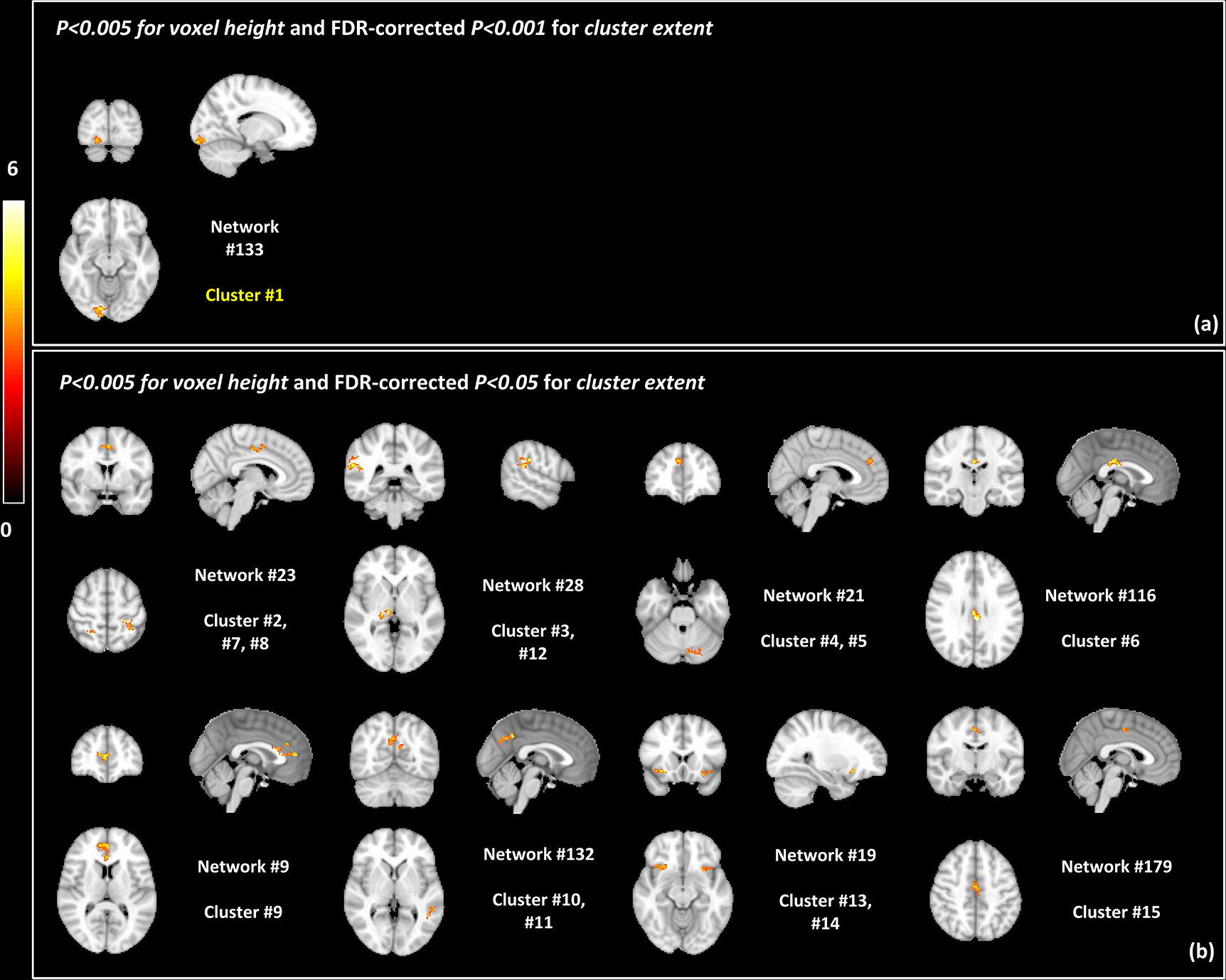

Supplement: S1 Fig — (a) Clusters #1 (P<0.005 for voxel height and FDR-corrected P<0.001 for cluster extent) (b) Cluster #2–15 (P<0.005 for voxel height and FDR-corrected P<0.05 for cluster extent). Clusters identified by both group sparse representation and tensor ICA are highlighted in color (color code shared with S2 Fig). (TIF) [file pone.0190097.s001.tif]

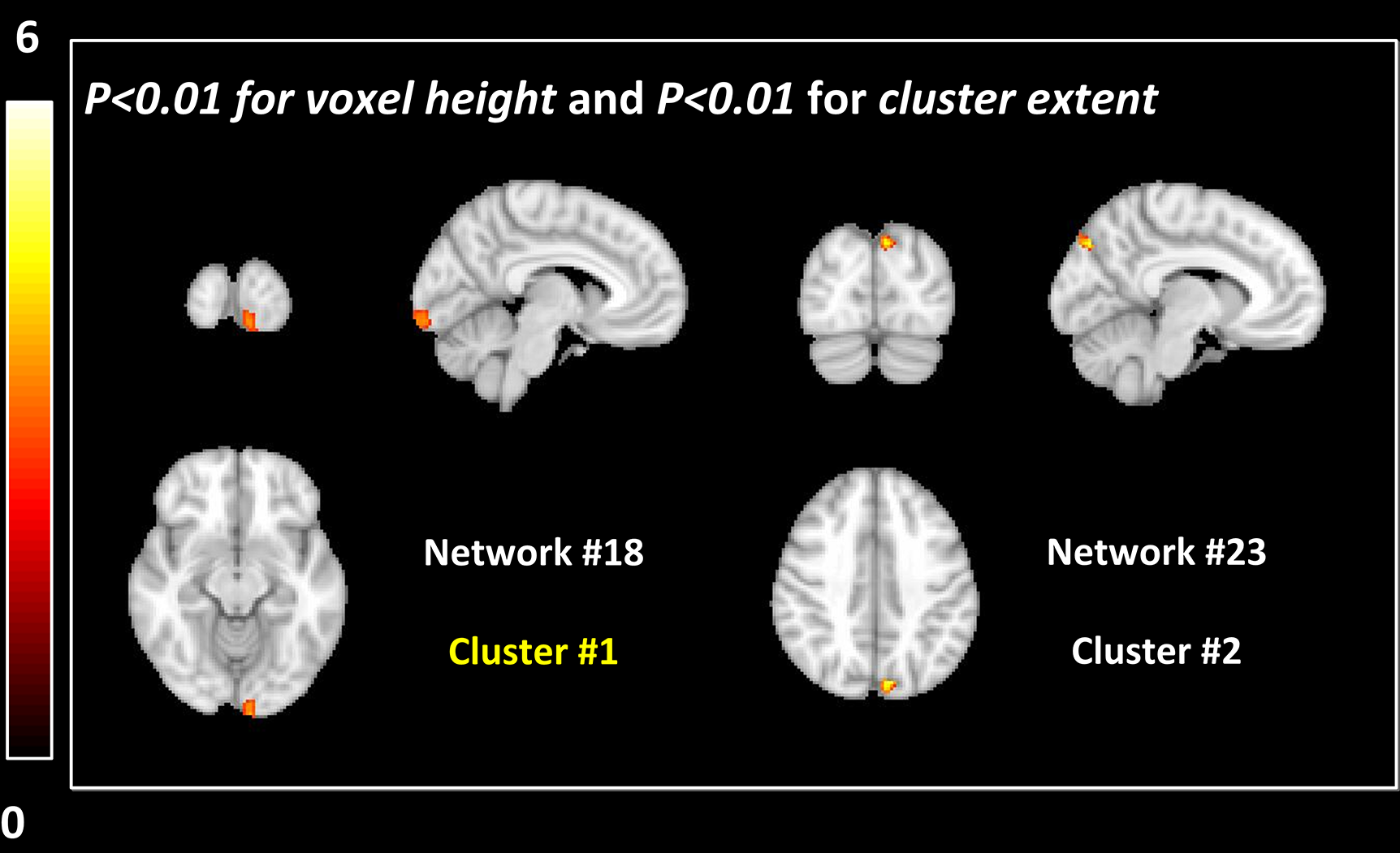

Supplement: S2 Fig — Clusters identified by both group sparse representation and tensor ICA are highlighted in colors (color code shared with S1 Fig). (TIF) [file pone.0190097.s002.tif]

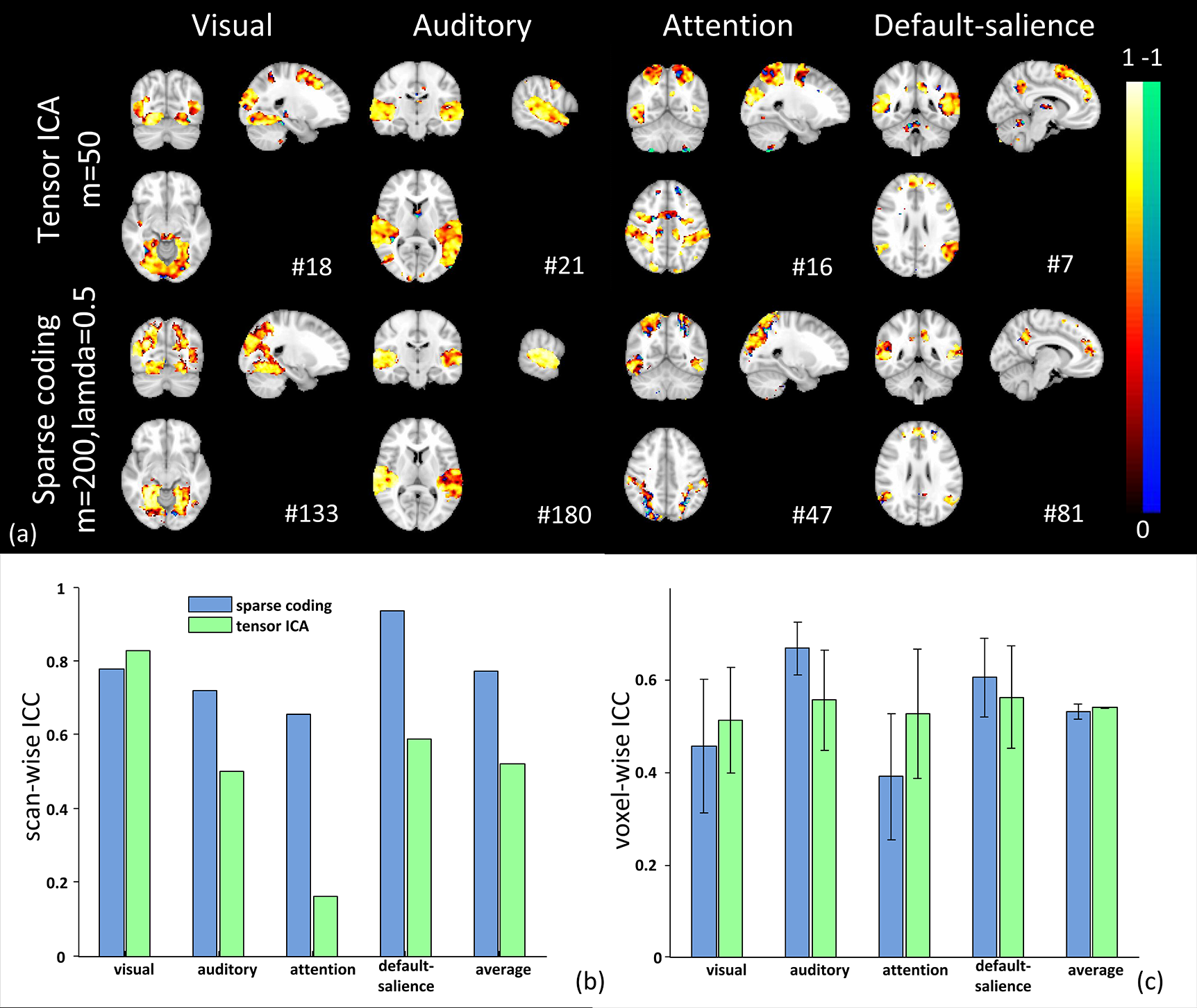

Supplement: S3 Fig — (a) Brain maps of the voxel-wise ICCs of matching networks identified by group sparse representation and tensor ICA for whole fMRI data. (b) scan-wise ICCs and (c) average voxel-wise ICCs of networks detected by both methods. Error bars signify variance. (TIF) [file pone.0190097.s003.tif]

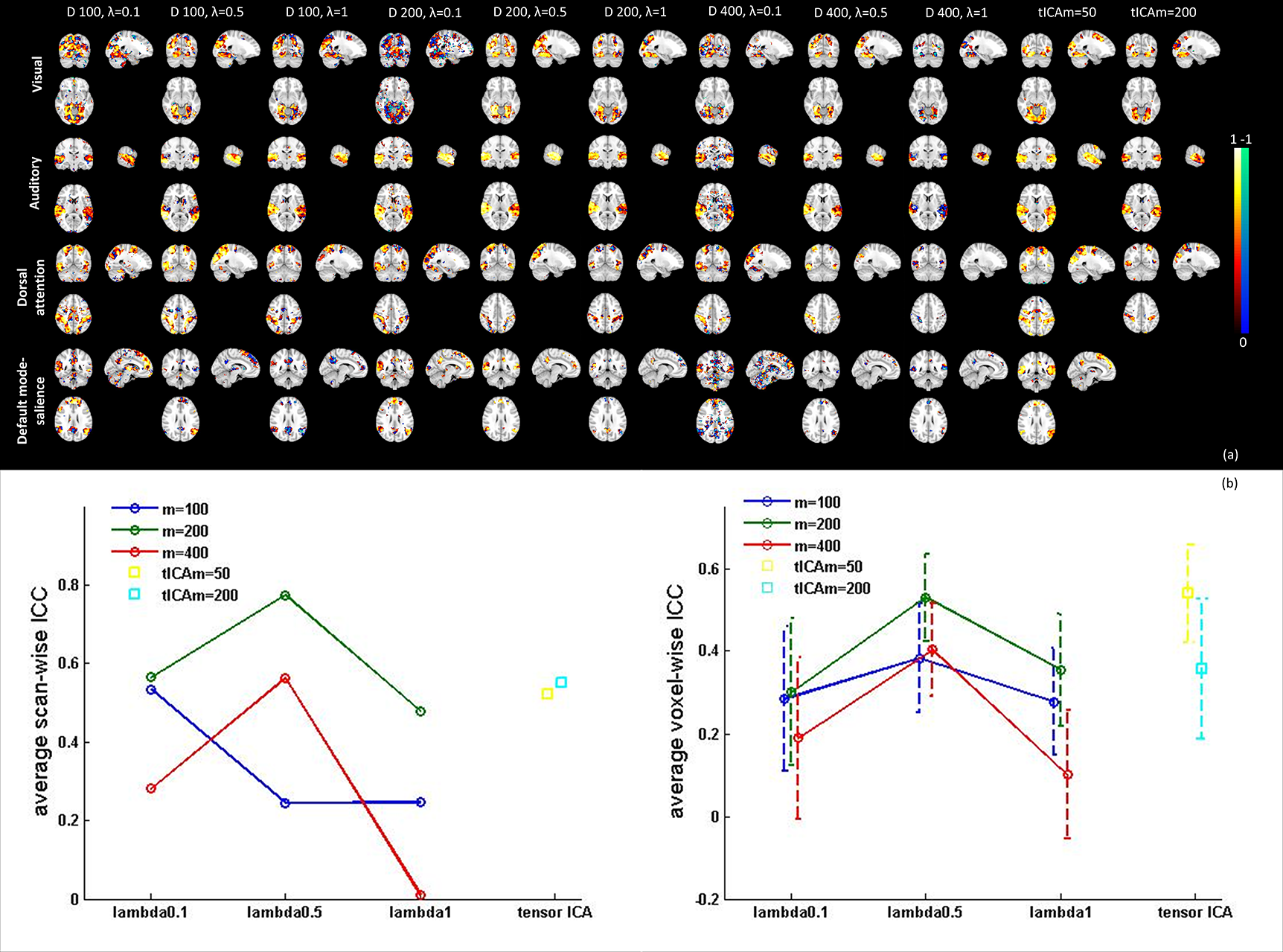

Supplement: S4 Fig — (a) Brain maps of the voxel-wise ICCs of matching networks identified by the two methods. (b) Average scan-wise and voxel-wise ICCs (Error bars signify the average variance of each network’s voxel-wise ICC). (TIF) [file pone.0190097.s004.tif]

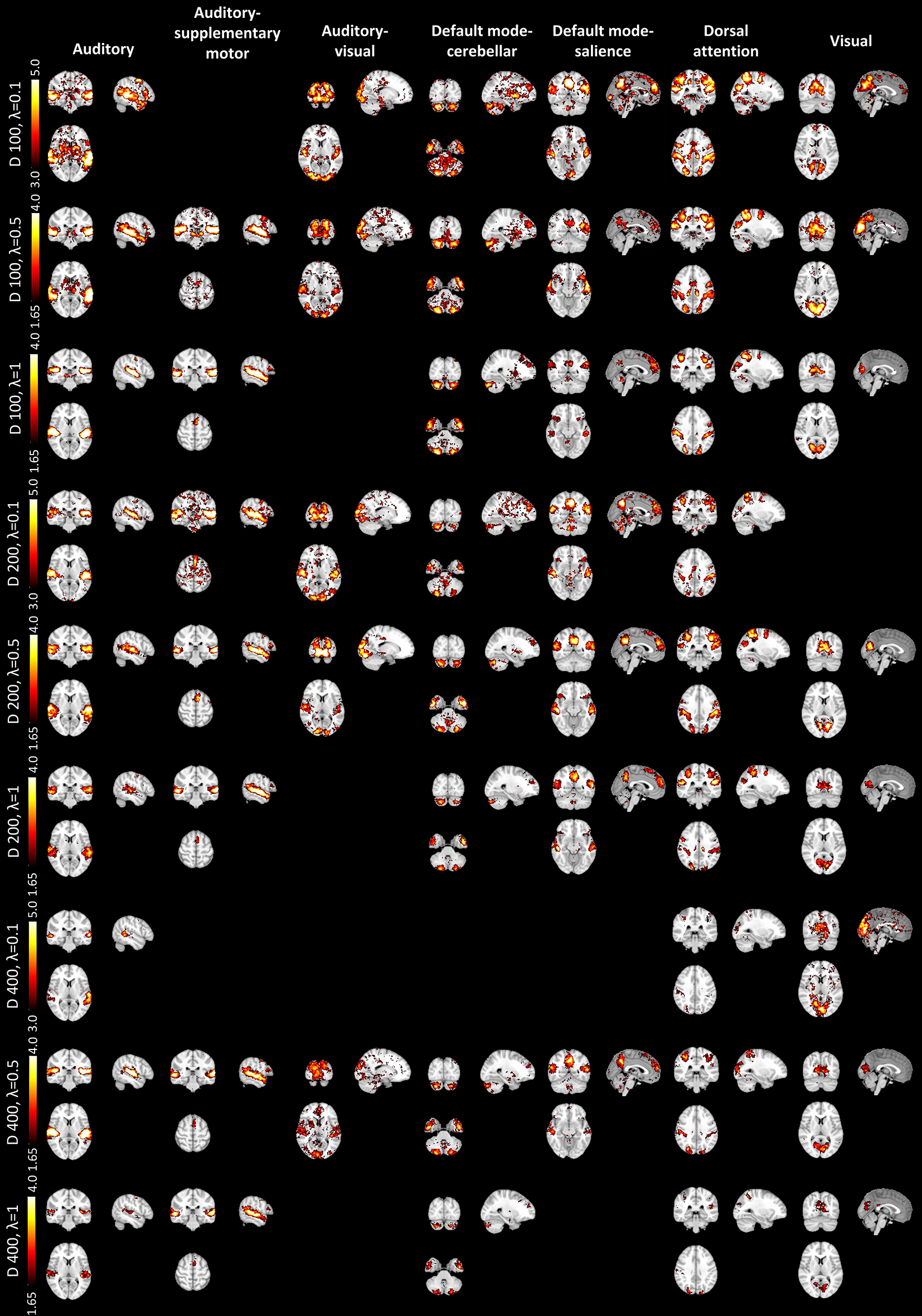

Supplement: S5 Fig — (TIF) [file pone.0190097.s005.tif]

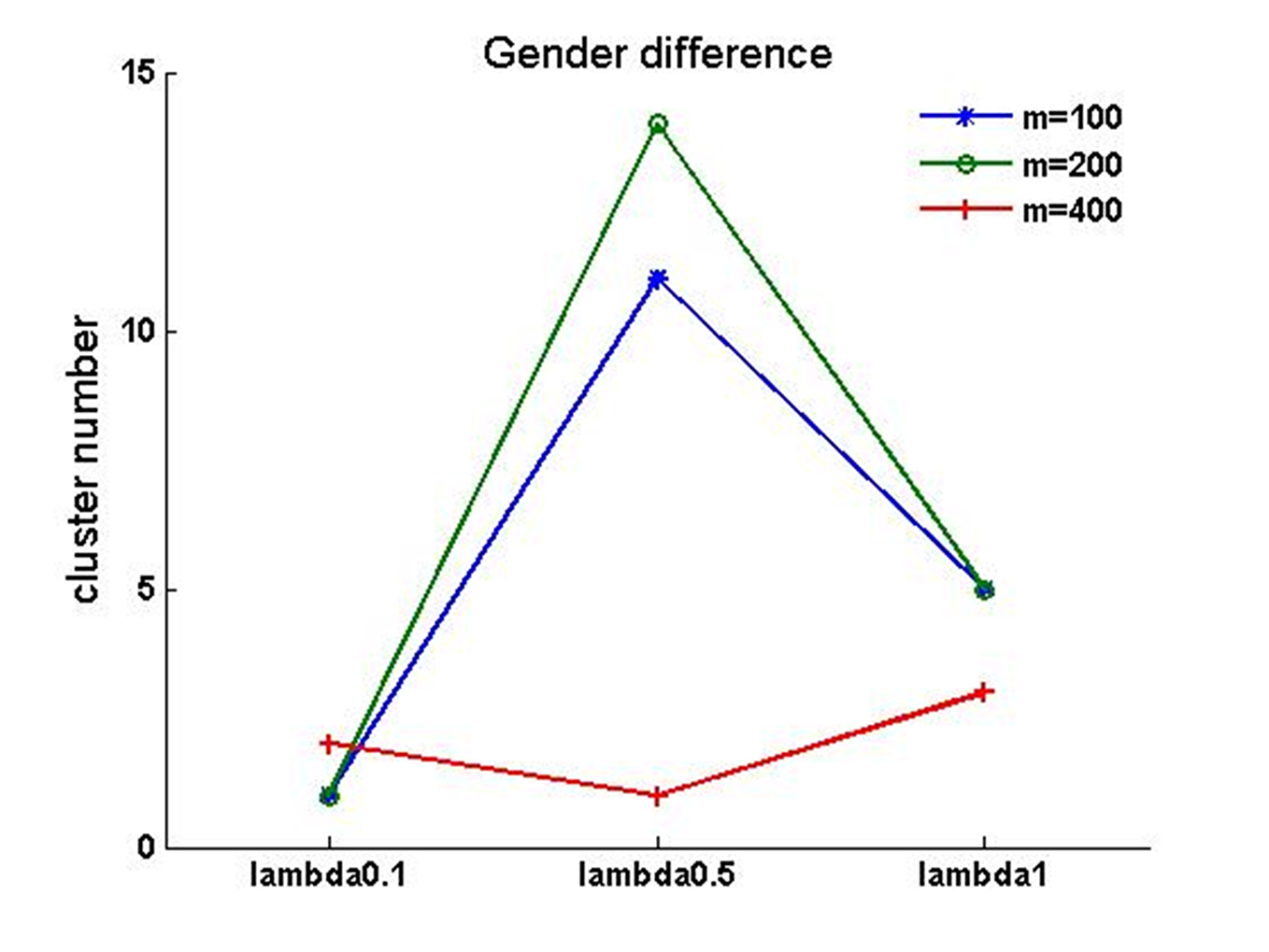

Supplement: S6 Fig — (TIF) [file pone.0190097.s006.tif]

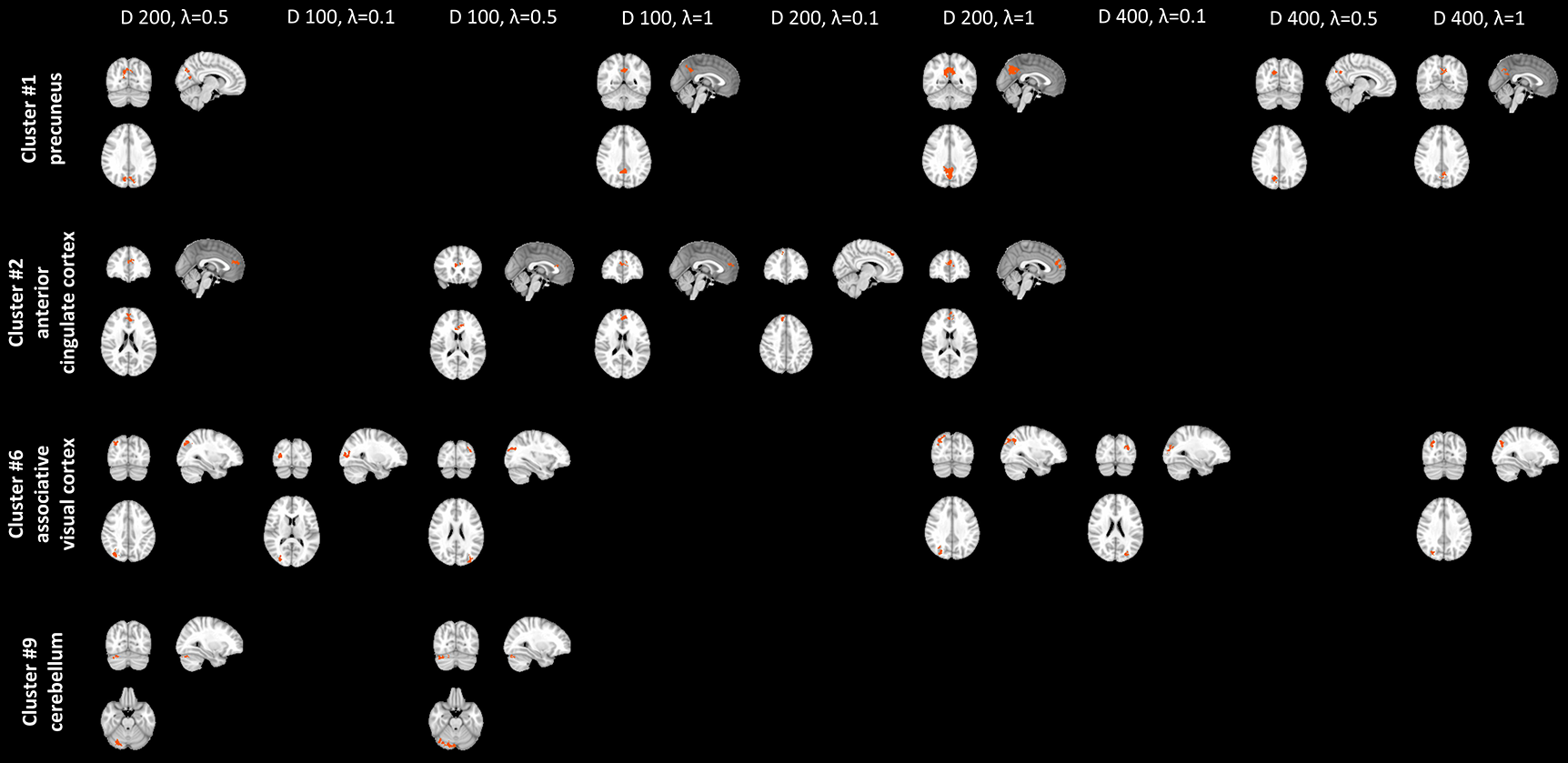

Supplement: S7 Fig — (TIF) [file pone.0190097.s007.tif]

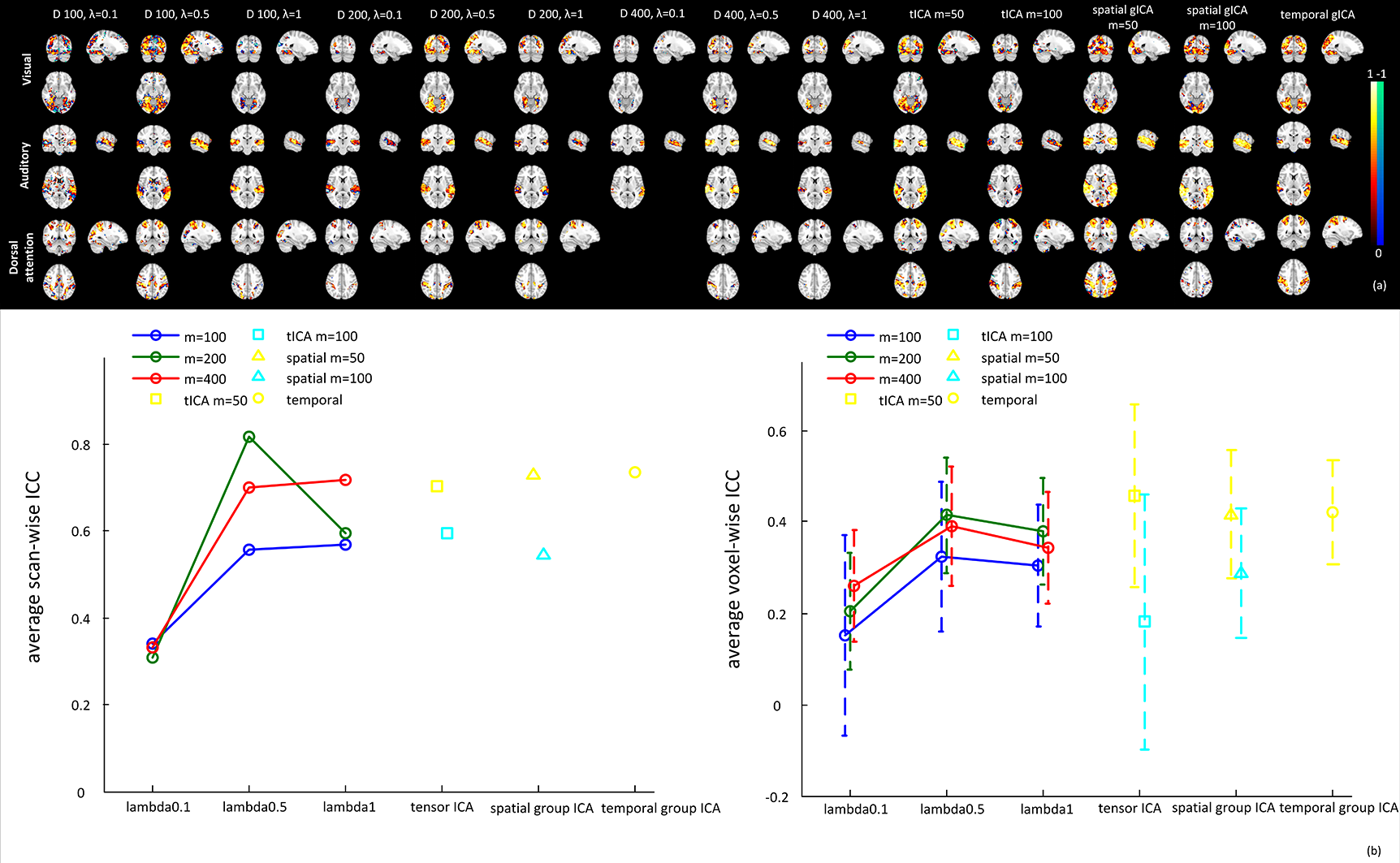

Supplement: S8 Fig — (a) Brain maps of the voxel-wise ICCs of matching networks identified by the three methods. (b) Average scan-wise and voxel-wise ICCs (Error bars signify the average variance of each network’s voxel-wise ICC). (TIF) [file pone.0190097.s008.tif]

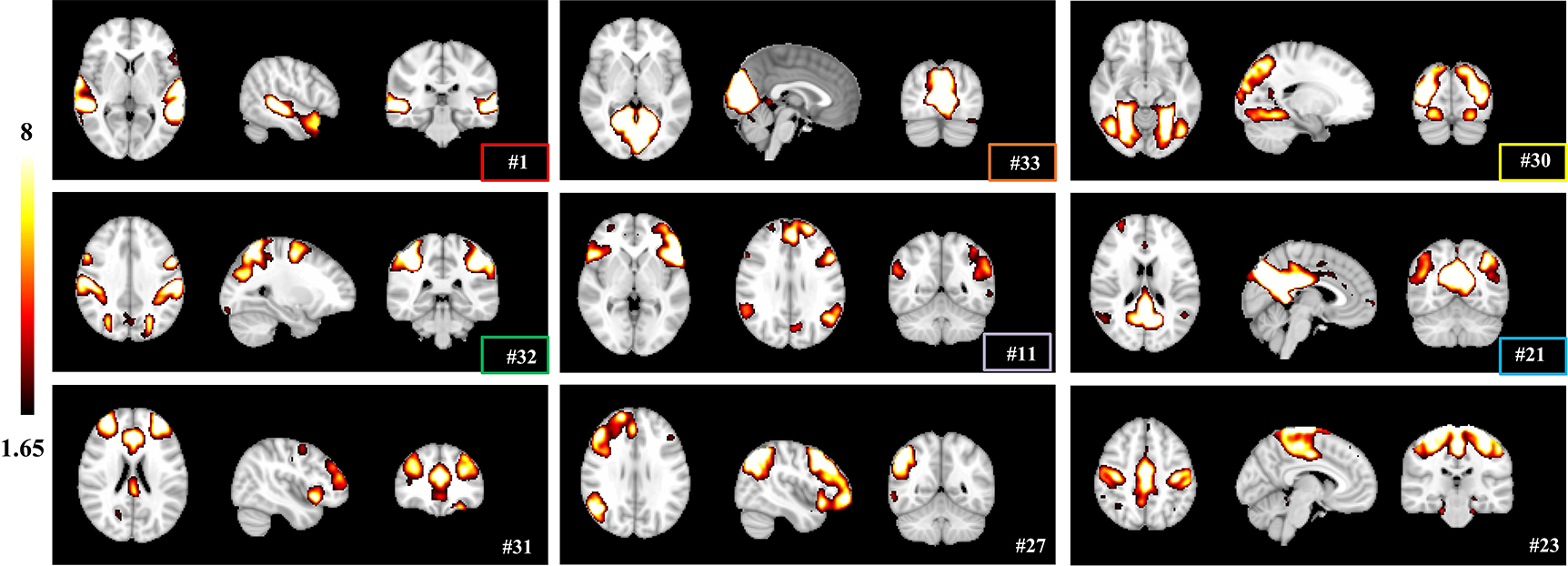

Supplement: S9 Fig — Networks identified by all methods are highlighted by rectangle frames (color code shared with Fig 3 in the main text). (TIF) [file pone.0190097.s009.tif]

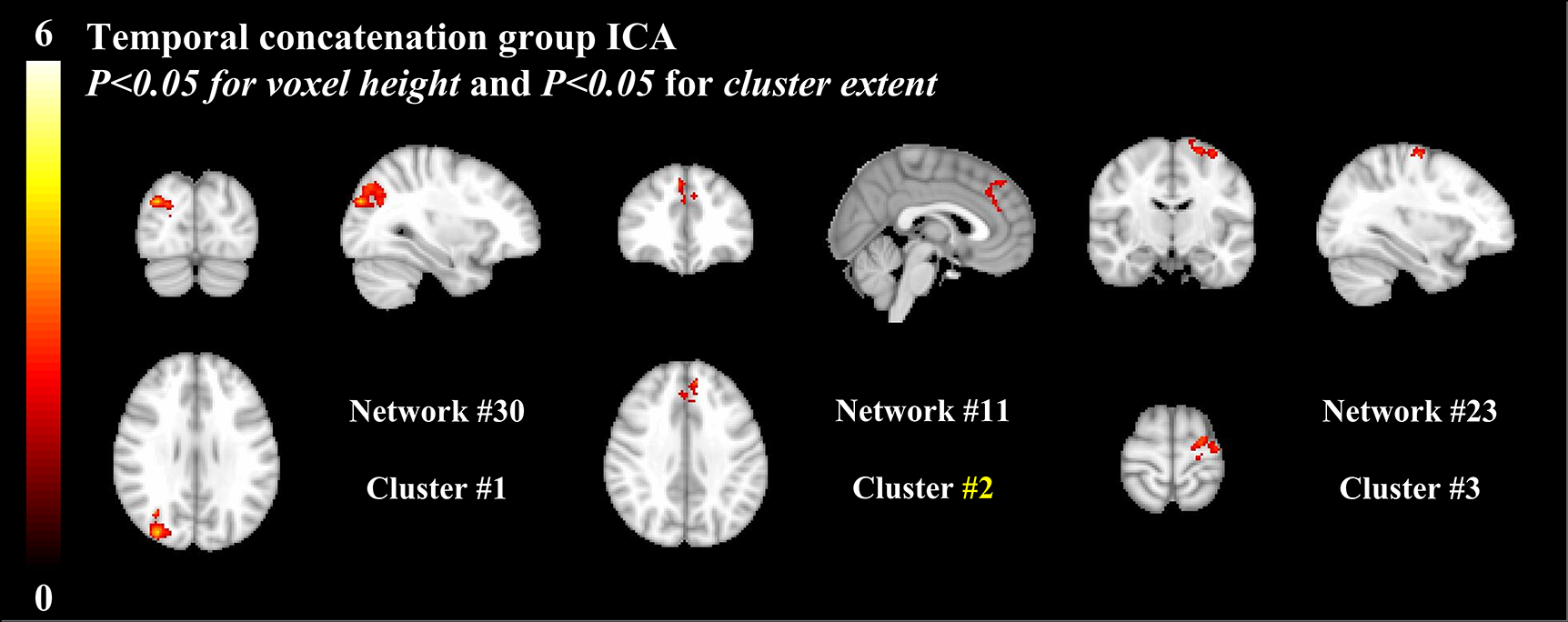

Supplement: S10 Fig — Clusters identified by all methods are highlighted in colors (color code shared with Fig 5 in the main text). (TIF) [file pone.0190097.s010.tif]

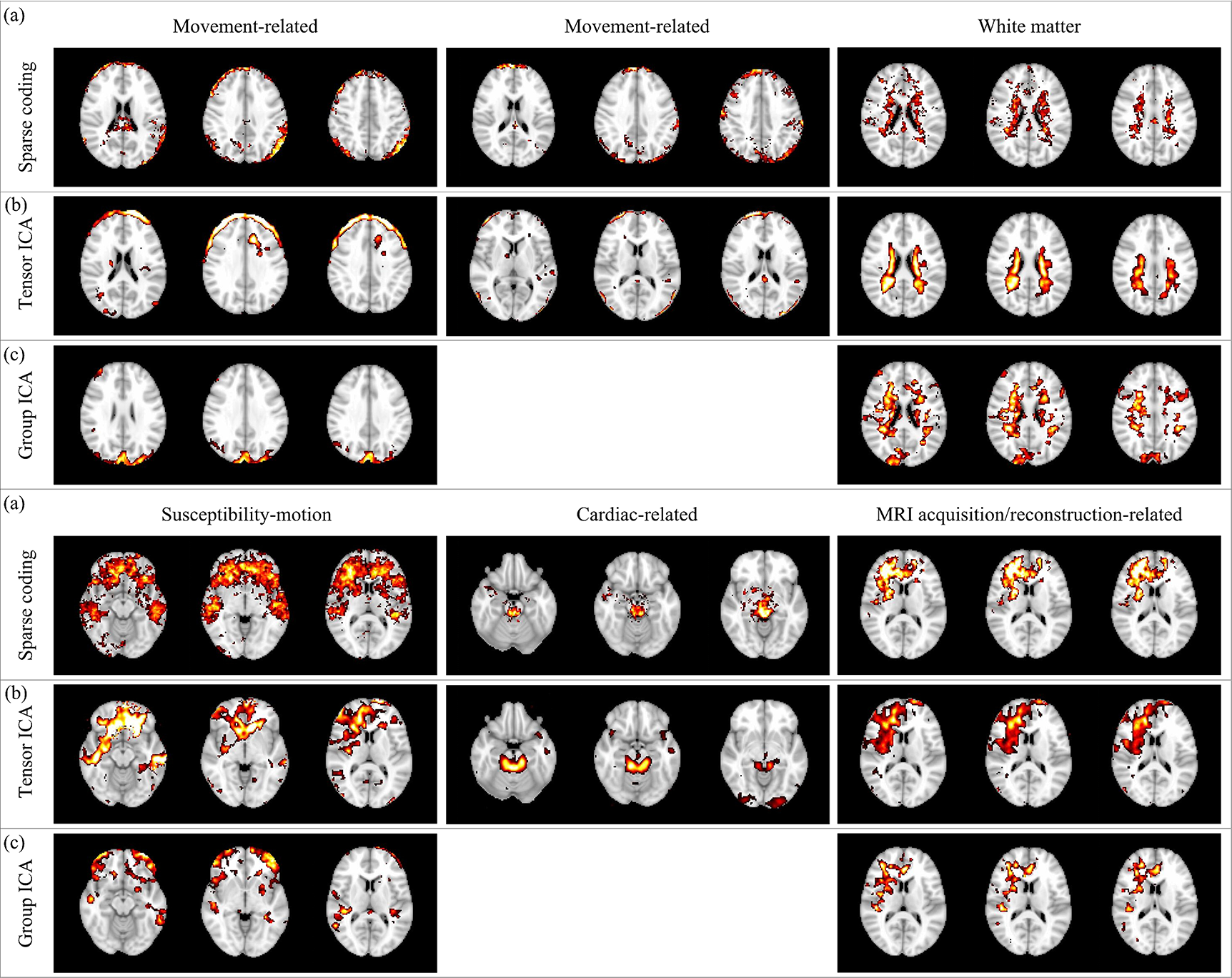

Supplement: S11 Fig — (TIF) [file pone.0190097.s011.tif]
