# Supplementary material for: Sparse coding reveals greater functional connectivity in female brains during naturalistic emotional experience
Source: PLoS One. 2017 Dec 22;12(12):e0190097. doi: 10.1371/journal.pone.0190097 (PMC5741239; doi:10.1371/journal.pone.0190097)
Supplement: S1 Table — (DOCX) [file pone.0190097.s012.docx]

**S1 Table. The variables and the explanations.**

| Variable | Explanation |
| --- | --- |
| *S*$\boldsymbol{\epsilon}\mathbb{R}^{\boldsymbol{t}\boldsymbol{\times}\boldsymbol{n}}$ | The aggregated matrix of whole brain fMRI signals from all the subjects. |
| D$\boldsymbol{\epsilon}\mathbb{R}^{\boldsymbol{t}\boldsymbol{\times}\boldsymbol{m}}$ | The dictionary matrix. |
| *A*$\boldsymbol{\epsilon}\mathbb{R}^{\boldsymbol{m}\boldsymbol{\times}\boldsymbol{n}}$ | The coefficient matrix. |
| *t* | Signal length. |
| *m* | Number of dictionary atoms. |
| *n* | Total number of signals. |
| *G_F_* | Female group. |
| *G_M_* | Male group. |
| *Fp (subscript)* | The *p*th subject in female group*.* |
| *Mq (subscript)* | The *q*th subject in male group*.* |
| *k* | The number of female subjects |
| *l* | The number of male subjects |
| *x (Subscript)* | label of subject*, Fp* or *Mq.* |
| $\boldsymbol{s}_{\boldsymbol{i}}$ | The $i$th sample in *S.* |
| $\boldsymbol{a}_{\boldsymbol{i}}$ | The $i$th coefficient column corresponding to $s_{i}$. |
| $\boldsymbol{d}_{\boldsymbol{i}}$ | The $i$th column of *D.* |
| $\boldsymbol{\lambda}$ | The regularization parameter of sparsity. |
