# Supplementary material for: Sparse coding reveals greater functional connectivity in female brains during naturalistic emotional experience
Source: PLoS One. 2017 Dec 22;12(12):e0190097. doi: 10.1371/journal.pone.0190097 (PMC5741239; doi:10.1371/journal.pone.0190097)
Supplement: S2 Table — (DOCX) [file pone.0190097.s013.docx]

**S2 Table. Brain regions with greater activation in females than males as detected by group sparse representation** **for whole fMRI data**(sorted by *p*-value in ascending order).

| Cluster  Index (P FDR-corrected) | (x y z) | T-value | Broadmann’s area | Region | Cluster size | Network Index |
| --- | --- | --- | --- | --- | --- | --- |
| 1 (0.001) | (14 -86 -12)  (14 -96 -14)  (18 -92 -6) | 5.16  4.82  4.41 | 17, 18 | Primary visual cortex, secondary visual cortex | 142 | 133 |
| 2 (0.008) | (-34 -44 60)  (-36 -32 62)  (-26 -42 64) | 4.8  4.23  4.16 | 3,5,40 | postcentral gyrus, superior parietal lobule | 130 | 23 |
| 3 (0.008) | (58 -28 28)  (60 -38 22)  (52 -36 12) | 6.75  5.44  4.11 | 13, 22, 40,42 | Superior temporal lobule, supramarginal, posterior insula | 252 | 28 |
| 4 (0.011) | (-6 -74 -28)  (-22 -72 -30)  (-28 -80 -30) | 4.07  3.69  3.62 |  | cerebellum crus 1, curs 2 | 108 | 21 |
| 5 (0.011) | (6 44 28)  (-4 50 26)  (-2 50 18) | 4.33  3.66  2.85 | 9,32 | superior medial frontal lobe, anterior cingulate cortex | 108 | 21 |
| 6 (0.015) | (-2 -26 30)  (2 -16 24) | 6.82  6.49 | 23 | middle and posterior cingulate cortex | 106 | 116 |
| 7 (0.022) | (26 -52 66)  (30 -52 54)  (16 -54 54) | 5.93  4.07  3.56 | 7,40 | superior parietal lobule, inferior parietal lobule, precuneus | 95 | 23 |
| 8 (0.022) | (10 -2 48)  (-2 2 48)  (6 -14 44) | 4.62  4.56  4.28 | 24,32 | anterior and middle cingulate cortex | 119 | 23 |
| 9 (0.023) | (4 48 8)  (0 34 26)  (2 30 10) | 5.08  4.64  4.08 | 10,32 | anterior prefrontal cortex, anterior cingulate cortex | 232 | 9 |
| 10 (0.026) | (-2 -52 40)  (-6 -64 20)  (4 -66 36) | 6.42  4.65  4.46 | 7,31 | precuneus, posterior cingulate cortex | 298 | 132 |
| 11 (0.026) | (-56 -42 4)  (-50 -46 10)  (-46 -52 0) | 4.75  3.16  3.12 | 21,22 | superior and middle temporal gyrus | 81 | 132 |
| 12 (0.035) | (8 -22 0)  (18 -24 0)  (22 -30 4) | 5.77  4.69  3.92 |  | thalamus | 87 | 28 |
| 13 (0.036) | (30 20 -10) | 5.29 | 13 | anterior insula | 76 | 19 |
| 14 (0.036) | (-28 16 -14)  (-40 16 -10)  (-26 26 -8) | 4.38  3.82  3.65 | 13 | anterior insula | 82 | 19 |
| 15 (0.042) | (0 -12 46)  (-8 -18 42)  (2 -4 42) | 4.37  3.96  3.55 | 24,31 | posterior and middle cingulate cortex | 121 | 179 |
