# Supplementary material for: Sparse coding reveals greater functional connectivity in female brains during naturalistic emotional experience
Source: PLoS One. 2017 Dec 22;12(12):e0190097. doi: 10.1371/journal.pone.0190097 (PMC5741239; doi:10.1371/journal.pone.0190097)
Supplement: S3 Table — (DOCX) [file pone.0190097.s014.docx]

**S3 Table. Brain regions with greater activation in females than males as detected by tensor ICA for whole fMRI data**(sorted by *p*-value in ascending order).

| Cluster  Index (P) | (x y z) | T-value | Broadmann’s area | Region | Cluster size | Network Index |
| --- | --- | --- | --- | --- | --- | --- |
| 1 (0.003) | (-8 -102 -12)  (-16 -96 -18) | 3.78  2.67 | 17,18 | primary visual cortex, secondary visual cortex | 111 | 18 |
| 2 (0.009) | (-8 -80 40)  (-6 -86 46)  (-16 -74 34) | 4.88  2.93  2.60 | 7,19 | cuneus | 74 | 23 |
