# Supplementary material for: Sparse coding reveals greater functional connectivity in female brains during naturalistic emotional experience
Source: PLoS One. 2017 Dec 22;12(12):e0190097. doi: 10.1371/journal.pone.0190097 (PMC5741239; doi:10.1371/journal.pone.0190097)
Supplement: S4 Table — (DOCX) [file pone.0190097.s015.docx]

**S4 Table. The questionnaire for subjects rating their experience after scanning session.**

**‘Neural Circuitry of Emotion Project’ Questionnaires**

**Name: Date:**

Have you seen this movie ‘Butterfly circus’ before? (yes/no)

Did you get bored during movie session?

Not at all: 1 2 3 4 5: very bored

How well did you enjoy movie session?

Not at all: 1 2 3 4 5: very enjoy

How emotional did you feel during movie?

Very sad: 1 2 3 4 5: very happy

How was the audio quality for movie?

Very poor: 1 2 3 4 5:very good

Is English your native language?

Yes No

How fluent is your English?

Very poor: 1 2 3 4 5: very fluent

How well did you understand the movie content? Please rate between 0 (not at all) and 100% (thoroughly): ______________
