# Supplementary material for: Sparse coding reveals greater functional connectivity in female brains during naturalistic emotional experience
Source: PLoS One. 2017 Dec 22;12(12):e0190097. doi: 10.1371/journal.pone.0190097 (PMC5741239; doi:10.1371/journal.pone.0190097)
Supplement: S5 Table — (DOCX) [file pone.0190097.s016.docx]

**S5 Table. Brain areas with greater activation in females than males as detected by temporal concatenation group ICA** (sorted by *p*-value in ascending order)**.**

| Cluster  Index | (x y z) | T-value | Broadmann’s area | Region | Cluster size | Network Index |
| --- | --- | --- | --- | --- | --- | --- |
| 1 | (32 -82 28)  (30 -76 44)  (34 -66 28) | 4.13  3.80  3.42 | 7, 19 | inferior parietal lobule, medial occipital gyrus | 466 | 30 |
| 2 | (-8 44 36)  (6 40 46)  (-8 26 42) | 3.27  3.05  2.89 | 6, 9, 32 | superior medial frontal lobe and anterior cingulate cortex | 259 | 11 |
| 3 | (-26 -6 66)  (-16 -16 76)  (-34 -14 66) | 5.63  3.89 | 6 | premotor cortex | 191 | 23 |
