# Supplementary material for: Sparse coding reveals greater functional connectivity in female brains during naturalistic emotional experience
Source: PLoS One. 2017 Dec 22;12(12):e0190097. doi: 10.1371/journal.pone.0190097 (PMC5741239; doi:10.1371/journal.pone.0190097)
Supplement: S6 Table — (DOCX) [file pone.0190097.s017.docx]

**S6 Table. Brain areas with greater activation in females than males as detected by tensor ICA** (sorted by *p*-value in ascending order)**.**

| Cluster  Index | (x y z) | T-value | Broadmann’s area | Region | Cluster size | Network Index |
| --- | --- | --- | --- | --- | --- | --- |
| 1 | (2 -62 32)  (0 -54 40) | 4.03  3.99 | 7,31 | precuneus, posterior cingulate cortex | 70 | 13 |
| 2 | (6 52 16)  (14 46 16) | 4.24  4.17 | 10 | superior medial frontal lobe and anterior cingulate cortex | 77 | 17 |
| 3 | (-10 -80 42)  (-16 -74 36) | 5.63  3.89 | 7, 19 | cuneus | 75 | 22 |
