# Supplementary material for: Sparse coding reveals greater functional connectivity in female brains during naturalistic emotional experience
Source: PLoS One. 2017 Dec 22;12(12):e0190097. doi: 10.1371/journal.pone.0190097 (PMC5741239; doi:10.1371/journal.pone.0190097)
Supplement: S7 Table — (DOCX) [file pone.0190097.s018.docx]

**S7 Table. Brain regions with greater activation in females than males as detected by spatial concatenation group ICA** (sorted by *p*-value in ascending order)**.**

| Cluster  Index | (x y z) | T-value | Broadmann’s area | Region | Cluster size | Network Index |
| --- | --- | --- | --- | --- | --- | --- |
| 1 | (-64 -10 4)  (-68 -20 6)  (-52 -22 -4) | 5.76  4.79  4.55 | 21, 22 | Superior and middle temporal gyrus | 365 | 3 |
| 2 | (28 -80 16)  (44 -70 6)  (48 -74 -4) | 4.94  4.23  3.87 | 19, 37 | Inferior and middle occipital gyrus | 270 | 3 |
| 3 | (64 -4 6)  (68 -8 12)  (66 -18 8) | 5.84  3.88  3.73 | 21, 22 | Superior and middle temporal gyrus | 208 | 3 |
| 4 | (10 54 14)  (6 50 8)  (16 50 2) | 5.09  4.30  3.69 | 10 | Superior medial frontal lobe and anterior cingulate cortex | 176 | 10 |
| 5 | (-24 -96 4)  (-20 -100 10)  (-28 -86 8) | 3.87  3.65  3.45 | 29, 30 | Cingulate cortex | 156 | 10 |
| 6 | (-56 -62 40)  (-52 -74 32)  (-48 -66 50) | 4.56  3.30  2.97 | 7, 31 | precuneus | 152 | 10 |
